# Facilitating rural access to quality health information through Little Free Libraries

**DOI:** 10.5195/jmla.2023.1585

**Published:** 2023-10-02

**Authors:** Jane Morgan-Daniel, Lauren E. Adkins, Margaret Ansell, Susan Harnett, Melissa L. Rethlefsen

**Affiliations:** 1 morgandanie.jane@ufl.edu, Community Engagement and Health Literacy Liaison Librarian, University of Florida Health Science Center Libraries, Gainesville, FL.; 2 lauren.adkins@ufl.edu, Pharmacy Liaison Librarian, University of Florida Health Science Center Libraries, Gainesville, FL.; 3 meansell@ufl.edu, Nursing and Consumer Health Liaison Librarian, University of Florida Health Science Center Libraries, Gainesville, FL.; 4 sharnett2022@gmail.com, Medical Librarian, Nemours Children's Health System, Jacksonville, FL.; 5 mlrethlefsen@gmail.com, Executive Director, Health Sciences Library & Informatics Center, University of New Mexico, Albuquerque, NM.

**Keywords:** Health Sciences Libraries, Public Libraries, Community Outreach, Community Engagement, Health Literacy, Consumer Health

## Abstract

**Background:**

In 2020 the Health Science Center Libraries (HSCL) at the University of Florida collaborated with the Okeechobee County Public library (OCPL) on their plan to install Little Free Libraries (LFLs) within their community. It was agreed that the HSCL would provide consumer health-related materials for the Little Free Libraries and training with the goal of improving health literacy, precision medicine, and increasing rural access to consumer health materials and services.

**Case Presentation:**

Using census data, the County Health Improvement Plan, and OCPL circulation data the team identified minority population groups, potential accessibility issues, and local consumer health information needs and barriers to select appropriate resources. Additionally, partnerships were created with the local Health Department, Parks and Recreation services, the Rotary Club, and other local organizations to make the project a success. A total of 424 books were selected for the LFLs and 40 unique online resources were selected, printed, and shipped to OCPL to be used during LFL reference sessions. Technology was purchased to assist OCPL with their planned community health reference outreach sessions. HSCL created and provided online training on facilitating consumer health outreach, conducting health information reference services, and promoting community engagement for OCPL.

**Discussion:**

LFLs have become an important resource for lower-income rural families in Okeechobee. There are 7 LFLs in Okeechobee County, with a goal of eventually establishing 15 total to provide vital health resources and books. Over 2,456 items have been circulated among the 7 LFLs since May 2020. Overall, the project has been successful with positive feedback received from the community and with OCPL planning to continue to expand the project.

## BACKGROUND

The Health Science Center Libraries (HSCL) at the University of Florida (UF) are committed to engaging with our local communities through active participation in community health organizations [1], direct outreach to the public at health fairs with general health information resources, and outreach on specific topics, such as HIV/AIDS [2]. In April 2019, HSCL employees were invited to participate in the UF Clinical and Translational Science Institute's Un-Meeting on Rural Health and Health Equity [3]. The Un-Meeting and further discussions with UF Health leadership prompted HSCL to consider ways to support statewide rural health.

In July 2019, the HSCL director attended the graduation of the Sunshine State Library Leadership Institute's cohort. During this event, one of the cohort, Sonja Chapa of Okeechobee County Public Library (OCPL), presented her leadership project, the beginnings of a broad effort to install Little Free Libraries (LFLs) throughout the rural county. Chapa planned to partner with local non-profit and county agencies to build 30 Little Free Libraries, which would be managed by OCPL. Little Free Library is an international nonprofit that fosters neighborhood book exchanges through outdoor book-sharing boxes [4]. There are hundreds of LFLs in Florida, but Okeechobee County did not have any until the OCPL project [5]. The new LFLs would be located on rural county properties with heavy foot traffic, such as parks and civic centers. While there has been criticism of LFLs and their impact on public libraries [6], others believe that libraries can engage with LFL stewards to prevent weeding bias and encourage equitable distribution of LFLs within a community [7,8].

A quick conversation about adding health-related materials to the Okeechobee LFLs to support rural health grew into a fruitful collaboration between HSCL and OCPL on a Network of the National Library of Medicine (NNLM) Southeastern Atlantic Region All of Us Community Engagement Project Award (January-September 2020). The project team aimed to improve rural residents' health literacy and understanding of precision medicine through a three-pronged approach:

- Purchasing/printing and distributing physical copies of authoritative consumer health materials, health literacy resources, and information about the All of Us Research Program
- Conducting train-the-trainer sessions for OCPL staff on consumer health information services; and
- Supporting weekly LFL-based health reference sessions

In this case report, we will describe the program's development and implementation, along with the impacts of COVID on the eventual outcomes.

## CASE PRESENTATION

### Understanding the Community

The first step in program development involved learning about local health needs and barriers to tailor resource selection for the LFLs to the demographics and health education needs of Okeechobee County residents. We used census data, the County Health Improvement Plan, and OCPL circulation data to identify minority population groups, potential accessibility issues, and local consumer health information needs. OCPL staff also met with their local health department, the County Health Educator, school nurses, and agricultural worker service organizations as agriculture is a primary business in the state [9]. Minority population groups were prioritized due health disparities, which are “preventable differences in the burden of disease, injury, violence, or opportunities to achieve optimal health that are experienced by populations that have been disadvantaged by their social or economic status, geographic location, and environment.” [10] Groups experiencing health disparities in the United States include racial and ethnic minorities, LGBTQ+ people, older adults, those with limited English proficiency, and people with disabilities [10,11].

Okeechobee is classed as entirely rural [12,13], and the planned LFLs were to be purposefully located in the most rural parts of the county which have the least access to public library services. Using census data, we identified a number of minority groups (Table 1). For this reason, we prioritized providing and promoting culturally and linguistically diverse consumer health resources for all ages, covering topics spanning from infant health to senior health.

**Table 1:** Census Population Estimates for Okeechobee County, Florida (total county population 41,537) [12]

**Hispanic or Latinx**	25.5%
**Black or African American**	9%
**Indigenous American**	1.6%
**Asian**	1%
**“Foreign Born”**	12.5%
**Speak a Language other than English at Home**	23.5%
**Elderly**	19.8%
**Veterans**	Approximately 7.2%

Facilitating access to both print and online health resources was imperative because census statistics revealed a large digital divide, as 22.9% of households did not own a computer and 41.9% did not have a home internet subscription. We targeted a 5th-8th grade reading level for resources, as 26.1% of county adults had not graduated high school. To maximize accessibility, we also prioritized including health information resources for the 10.8% of residents under 65 who are disabled [13]. We identified health topics for LFL resources in alignment with the county's 2016 Community Health Improvement Plan [13], which identified local public health issues (Table 2) and three health education priorities: nutrition, disease prevention, and navigating the healthcare system. Circulation data from OCPL reflected similar local concerns: the most frequently borrowed health books in 2019-2020 (as reflected by subject headings and book titles) related to healthy eating, chronic pain, mental health, ADHD, menopause, diabetes, obesity, smoking cessation, autism, cancer, and heart disease (see project materials https://doi.org/10.17605/OSF.IO/HJM9U). Prior to this project, according to the circulation statistics from OCPL 2017-2020, the total number of health-related materials circulated was 130 items.

**Table 2:** Health Issues Highlighted in County Health Improvement Plan [13]

**Depression**	18.9% of county adults versus approximately 16% at the state level
**Heavy or Binge Drinking**	21.6% of county adults versus a state rate of 17.6%
**Chronic Obstructive Pulmonary Disease, Emphysema, or Chronic Bronchitis**	17% of county adults versus 7.4% for the state
**Stroke**	7% of county adults versus 3.7% at state level
**Diabetes**	Age-adjusted rate hospitalizations from or with diabetes was 4,509.8 versus 2,321.2 for the state*
**Teenage Births**	Rate of 21.1 versus 7.3 for Florida overall
**Sexually Transmitted Diseases**	Rate of chlamydia, gonorrhea, and infectious syphilis was 501.4 per 100,000 in 2015 versus Bacterial Sexually Transmitted Diseases at 613.1 per 100,000 for Florida overall*

* Total County Population of 41,537

## SELECTING AND PURCHASING RESOURCES

### Book Selection

A total of 424 books were selected for the LFLs and all purchase decisions were guided by community demographics and health needs (see Data Availability Statement). We selected health books in English and Spanish, with content and images tailored for racially diverse audiences and with diverse and positive representations of people identifying as disabled, LGBTQ+, veterans, rural-residing, and caregivers wherever possible. Material was written for a wide range of ages, including a number of children's books, which were most likely to have pictures, use plain language, and be written at lower reading grade levels.

### Online Resources

Forty unique online consumer health and health literacy resources were selected, printed, and shipped to OCPL to be used during the intended LFL reference sessions (see Data Availability Statement). Resources included bookmarks, brochures, posters, and wallet cards created by a variety of governmental and non-governmental organizations, such as the American Heart Association, California Department of Public Health, and NNLM. Printed resources in English and Spanish were ordered where possible. In addition to the health topics noted above, the materials covered other topics, including All of Us, citizen science, health information evaluation, libraries and community health, MedlinePlus, and precision medicine (All of Us is a Research Program of the National Institutes of Health that aims to build a nationwide dataset of volunteers to enable individualized health prevention, treatment, and care). A print subscription to the NIH MedlinePlus Magazine was also organized.

### Technology and Charter Signs

We also purchased a portable printer, four Chromebook laptops, and two Wi-Fi hotspots with unlimited data plans to assist OCPL with their community health reference outreach sessions which were intended to take place at the LFL locations across the county. Additionally, we registered the LFLs with littlefreelibrary.org, receiving charter signs and enabling our LFLs to be added to the online Little Free Library World Map [5].

## DEVELOPING AND CONDUCTING TRAINING

Though public libraries are often the first stop for consumers seeking health information, public librarians often feel unqualified to answer health-related questions [14], to evaluate online sources, and to navigate medical databases [15]. Librarian discomfort and unfamiliarity with health resources may create barriers for patrons seeking information, while librarians may be frustrated with their inability to provide patrons with up-to-date, authoritative sources due to lack of knowledge [16]. Thus, a significant project objective was ensuring that OCPL staff and local county health department educators were equipped to provide consumer health information services, which requires an understanding of health literacy, the ability to select and evaluate consumer health resources, and effective patron communication and health reference skills. To achieve this objective, HSCL librarians employed a train-the-trainer (TTT) model of disseminating health information and resources. TTT is an effective method of preparing workshop participants to deliver information to others, because training others to disseminate information eliminates gatekeeping, broadens distribution, and leads to long-term sustainability of programs [17].

The training was initially designed to take place over two consecutive days in person at OCPL. Due to the pandemic, OCPL closed to the public and HSCL librarians transitioned the sessions to a virtual format, given over one full day via Zoom. Participants in the live session included eight members of OCPL staff, and the training recording was circulated to the Okeechobee County Health Department and the Lake Okeechobee Rural Health Network.

We designed the training to lead participants from theory to practice, with the *Eight Core Competencies for Providing Consumer Health Services* from NNLM in mind [18]. The Medical Information Services Librarian led the morning session with an introduction to consumer health information topics. Participants first learned how to critically evaluate online information using Health on the Net (HON) principles, including authority, complementarity, privacy, and transparency. As a group, participants then evaluated health websites that patrons might find in a Google search. Authoritative consumer health websites such as MedlinePlus, Pillbox, Genetics Home Reference, and Clinical Trials.gov were demonstrated. Next, the Community Engagement and Health Literacy Librarian introduced the concept of health literacy and its impact on healthcare and gave tips for identifying and communicating with patrons with limited health literacy. Lastly, the ethical, legal, and privacy concerns of conducting health reference interviews were discussed.

The afternoon session focused on issues related to potential library programming. The Consumer Health Librarian covered marketing and promoting health outreach events, best practices for tabling at events, finding and developing community partnerships, and using social media to promote library events. The second half highlighted citizen participation in research, the Pharmacy Librarian described the NIH All of Us program and provided resources to promote community engagement with this program and participants also learned about citizen science and how to get involved with projects in their area.

## EVALUATION

### Evaluation Methods

Efficacy of the TTT session was assessed by administering the validated eHealth Literacy Scale (eHEALS) instrument before and after the training via Qualtrics (approved exempt by University of Florida Institutional Review Board). eHEALS is an eight-item validated instrument which measures “…consumers' combined knowledge, comfort and perceived skills at finding, evaluating and applying electronic health information to health problems” [19]. We also administered a short participant satisfaction survey provided by NNLM.

As part of the ongoing internal usage assessment of the LFLs, OCPL staff make regular rounds of the installed LFLs to restock them with print health information materials as necessary. They keep a log of which LFL locations need to be restocked most frequently and with which materials. This information is used to assess the popularity of the various LFL locations and health information topics, which in turn informs OCPL health-related programming and outreach events.

### Evaluation Results

**Figure 1:**
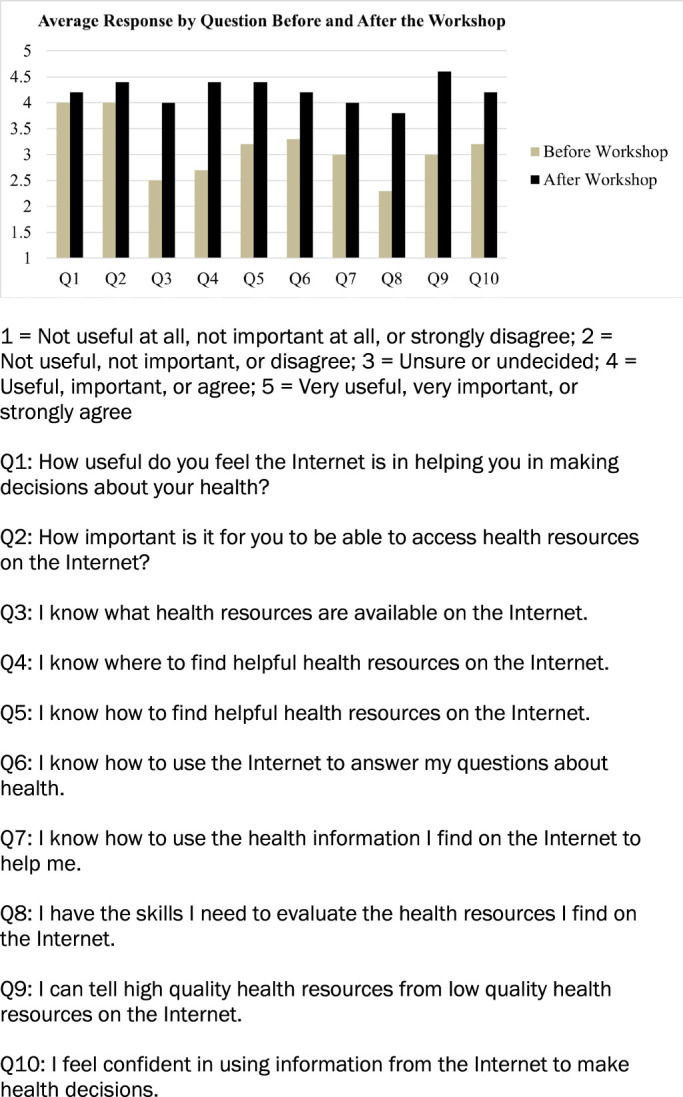
eHEALS Average Response by Question Before and After the Workshop

Six participants took the voluntary eHEALS survey before the workshop and five completed it afterwards (Figure 1). Despite this discrepancy, the overall eHEALS results demonstrate that the workshop successfully increased participants' perceived ability to navigate health information online (from 3.1 (unsure/undecided) to 4.2 (useful/important/agree) out of a possible 5). Additionally, answers for each of the 10 questions showed improvement, with the score for Question 4 (“I know where to find helpful health resources on the internet”) increasing by 34% from 2.7 to 4.4, Question 9 (“I can tell high quality health resources from low quality health resources on the internet”) increasing by 32%, and Question 3 (“I know what health resources are available on the internet”) and Question 8 (“I have the skills I need to evaluate the health resources I find on the internet”) both increasing by 30%.

### Reference Sessions

The project's intended third component was for OCPL staff who attended the training sessions and County Health Department staff who watched the training videos to host regular health reference sessions at Little Free Library locations. Due to COVID-19, this aspect of the program was not implemented during the project time frame, though OCPL staff were able to use their acquired skills during temporary live virtual health reference sessions and through reader's advisory services for health materials for their patrons; this has provided a positive alternative use for grant-funded technology and materials. In addition, the Wi-Fi hot spots, originally purchased for providing reference sessions at the LFLs, became an important component of the OCPL's COVID-19 strategy. Based on their experience with the LFL hot spots, the OCPL purchased 148 hot spots for patrons to check out by mid-2020 [20], and in 2021, the city installed seven new Wi-Fi hot spot towers in the city park, many of which were located near LFLs. [21]

## IMPACT

From the very beginning, this project was intended to empower OCPL to engage in consumer health information services and to kickstart their efforts by providing training and resources. To that end, OCPL staff are now equipped to host weekly in-person reference sessions at various LFL locations around the county as part of their outreach efforts. The technology provided supports immediate access to trustworthy and culturally relevant consumer health resources; library staff can search for, access, and print out resources to answer patron questions during these sessions. Staff also utilize these resources to support virtual reference, ensuring that all patrons can access library assistance, regardless of their location or ability to travel. OCPL staff continue to collect data on reference transactions and material use statistics and use these data to determine which LFL locations merit permanent Wi-Fi hotspots, regular library outreach services, and to schedule health information programming.

The LFLs have become an important resource for lower-income rural families in Okeechobee. OCPL successfully markets the LFL and virtual reference sessions to their community members through physical and digital flyers, local news outlets, and social media. There are currently 7 LFLs in Okeechobee County, with a goal of eventually establishing 15 total. Another indicator of community impact is the high usage of items from the LFLs. As of March 2022, over 2,456 items have been circulated among the 7 LFLs. Feedback from OCPL staff highlights the impact of this collaboration. The Library Director commented via email, “the books you sent us were amazing and much needed…this grant has been a blessing for our community” [22]. The LFLs are checked monthly by OCPL staff when they inspect the LFLs for wear and tear. The staff takes note of how many health resources have been taken from the LFLs, which is their basis for evaluation and approximate circulation statistics. The OCPL librarians monitor and restock health resources monthly. Any materials contributed to the LFLs by other community members are kept for circulation. While recognizing that quality control of patron-added health information is a likely issue, the public library believes that all additional materials are valuable for attracting more users because they reflect the community's interests. The popularity and patron-centeredness of the LFLs is therefore evident.

Since the end of the project, OCPL has begun a new Mobile Library Van service with the goal of incorporating all of the technology and resources provided by the original project. This includes using the laptops and printer to provide technical support and adding health reference services to the list of librarian services offered via the van.

An unanticipated finding of this project was the community's need for internet hotspots and access to wireless internet services in the Okeechobee area. Over 100 hotspot devices have been added to the OCPL circulating collection, and OCPL's entire library cooperative, which includes seven additional library branches in the surrounding five counties, launched a copycat lending service. In hindsight, this finding reflects the need identified by Real et al. that rural libraries have for technological support [23]. OCPL strengthened their partnership with the local Health Department and partnered with them to facilitate COVID-19 vaccinations, sponsor blood drives, and regularly host joint health programs. This included a Heart Health event in February 2022 that provided free blood pressure screenings for the community at OCPL.

OCPL also continues to maintain relationships with other local community partners to support their health information services, having worked with the local Rotary Club and the county Parks and Recreation department to create a StoryWalk to attract local families to the LFL locations [24]. StoryWalk is an international project in which laminated pages from children's books are installed along local outdoor trails using wooden stakes. This StoryWalk was designed by OCPL to promote health and literacy through active, mobile engagement. The OCPL team also presented their health information services, as well as information about the All of Us research program, at a local gathering of non-profit community-focused organizations.

The relationship between HSCL and OCPL also continues; HSCL librarians are available to provide virtual and telephone support to assist with complex health reference questions received by OCPL that may benefit from our additional resources and expertise. HSCL librarians also keep OCPL staff up to date about new sources of freely available authoritative health information relevant to their needs.

## BARRIERS AND FACILITATORS

The biggest challenge faced was the onset of COVID-19 halfway through the project period, impacting multiple aspects of the project. This included supply chain issues that delayed the purchase of consumer health print resources and technology from several vendors. Additionally, state pandemic-related restrictions did not permit state employee business travel, preventing the HSCL librarians' travel to Okeechobee to lead the planned in-person training sessions. The workshop was instead conducted virtually. These restrictions also impacted the building and installation of LFLs. One of the benefits of this change was that the funding allotted for travel was repurposed to purchase additional print resources and to cover increased technology costs.

Since outreach events and in-person reference services were delayed, virtual health reference sessions were offered by OCPL through phone, email, and online meetings, to accommodate different technological skill levels of patrons. One of the lessons learned was to order necessary materials early on to provide time to respond to unanticipated delays, especially when facing expenditure deadlines. Nevertheless, one of the positive aspects of COVID-19 was that it emphasized the importance of accessible quality health information, the value of the public library as a community resource and of multi-institutional partnerships for mutual support, and the need for consumer health information resources and services in underserved communities.

## IMPLICATIONS FOR PRACTICE

Multi-institutional partnerships are an essential strategy in expanding the capacity and reach of library efforts, particularly in rural areas. HSCL's community engagement efforts rely on public partnerships, as the library itself has very little direct interaction with the public and has successfully partnered with a variety of institutions for previous community engagement projects [1,2]. Academic health sciences libraries can offer support in pursuing grant funding and continuity during staff transitions, challenges that rural libraries often face when conducting outreach programs [25,26].

Communication continues to be an essential part of multi-institutional projects. At the outset, project team members should share their communication preferences and any specific availability issues. The growing use of video conferencing technologies during the pandemic made remote synchronous meetings much simpler and more accessible than in the past, but these can still be challenging to schedule; regular check-in meetings are key to keeping the entire team up to date with changes and giving everyone a voice in the project. Setting clear expectations for responsiveness for asynchronous communications like email is also important, particularly as deadlines approach. Finally, team members should be willing to utilize multiple means of communication, including something as simple as picking up the telephone, if that is what best fits the team's work schedule and communication preferences.

Another element of multi-institutional project teams, particularly institutions which have not worked together before, is the importance of getting to know one another and learning about each other's work context, skill sets, and experience. This builds trust and demonstrates that each institutional partner has something unique to offer. Clearly outlining the role of each institution and allowing each team member to take ownership of the aspect of the project that was most engaging to them sets the project up for success. In this project, understanding the context of the OCPL was essential to the work, both in terms of knowing the health information needs of the community and also in understanding the health information skills of OCPL staff. To that end, continuous OCPL staff input and reviewing community health assessments was a crucial part of the project's activities.

## CONCLUSIONS

While this project faced challenges unique to the pandemic as well as challenges common to multi-institutional collaborations, it has successfully achieved its aim of establishing health information services at OCPL, as well as improving rural residents' awareness of and access to authoritative consumer health resources. Additionally, the project built and strengthened new collaborations with community partners, revealed the need for new services, and based on the circulation statistics is highly valued by the members of the community. Since collaborating with HSCL the OCPL has continued partnering with local organizations to promote and provide health information resources to the community. Recent partnerships have formed with the Florida Community Health Centers, Healthy Start of Okeechobee, Okeechobee County Senior Services, and the Treasure Coast Food Bank Community Health Advocate. Overall, the innovative use of LFLs as a library outreach tool, combined with the incorporation of health resources and reference services, is contributing towards the reduction of health information disparities in underserved populations, as county residents are empowered to make effective use of information for health decision-making.

## Data Availability

Data associated with this article are available in the Open Science Framework at https://doi.org/10.17605/OSF.IO/HJM9U.

## SUPPLEMENTAL FILES

**Appendix A:** Print Books Purchased

**Appendix B:** Online Resources for Printing and Ordering

**Appendix C:** Average Response by Question

**Appendix D:** eHeals Results
