# Supplementary material for: Facilitating rural access to quality health information through Little Free Libraries
Source: J Med Libr Assoc. 2023 Oct 2;111(4):811–8. doi: 10.5195/jmla.2023.1585 (PMC10621719; doi:10.5195/jmla.2023.1585)
Supplement: Supplementary file 1 — Appendix A: Print Books Purchased [file jmla-111-4-811-s01.pdf]

Appendix A: Print Books Purchased

Title	Authors	Year published
15 THINGS THEY FORGOT TO TELL YOU ABOUT AUTISM: THE STUFF THAT TRANSFORMED MY LIFE AS AN AUTISM PARENT.	ELLEY, DEBBY	2018
#chill: Turn off your job and turn on your life	Robinson, BE	2019
(DON'T) CALL ME CRAZY: 33 VOICES START THE CONVERSATION ABOUT MENTAL HEALTH.	JENSEN, KELLY	2018
¿Qué hacer? Seven book series, five of which are about children's health	Gloria Mayer and Ann Kuklierus	2012
¿Qué hay dentro de mi?, six book series	Dana Meachen Rau	2007
100 QUESTIONS & ANSWERS ABOUT CHRONIC PAIN.	MALETIC, VLADIMIR and University of South Carolina	2012
100 QUESTIONS & ANSWERS ABOUT HIV AND AIDS.	GALLANT, JOEL E and John Hopkins University	2017
100 QUESTIONS & ANSWERS ABOUT KIDNEY CANCER.	CAMPBELL, STEVEN C and Cleveland Clinic	2019
100 QUESTIONS & ANSWERS ABOUT LIVER CANCER	ABOU-ALFA, GHASSAN K and MEMORIAL SLOAN-KETTERING CANCER CENTER.	2020
100 QUESTIONS & ANSWERS ABOUT LUNG CANCER.	SCHILLER, J.H. and U. OF TEXAS SOUTHWESTERN MEDICAL CENTER	2014
100 QUESTIONS & ANSWERS ABOUT OVARIAN CANCER	DIZON, DON S. HARVARD MEDICAL SCHOOL	2016
100 QUESTIONS & ANSWERS ABOUT PROSTATE CANCER.	ELLSWORTH, PAMELA. UNIVERSITY OF CENTRAL FLORIDA COLLEGE OF MEDICINE	2019
100 QUESTIONS & ANSWERS ABOUT YOUR CHILD'S TYPE 1 DIABETES	PLATT, ELIZABETH	2012

Title	Authors	Year published
12-WEEK DIABETES COOKBOOK: YOUR SUPER SIMPLE PLAN FOR ORGANIZING, BUDGETING, AND COOKING AMAZING DINNERs	GASSENHEIMER, LINDA	2018
15-minute diabetic meals	Hughes, Nancy S.	2010
21 THINGS YOU NEED TO KNOW ABOUT DIABETES AND WEIGHT-LOSS SURGERY.	CUNNEEN, SCOTT A	2017
21 THINGS YOU NEED TO KNOW ABOUT DIABETES AND YOUR HEART.	WEISENBERGER, JILL	2015
36-HOUR DAY: A FAMILY GUIDE TO CARING FOR PEOPLE WHO HAVE ALZHEIMER DISEASE, OTHER DEMENTIAS, AND MEMORY LOSS.	MACE, NANCY L	2017
4-INGREDIENT DIABETES COOKBOOK: SIMPLE, QUICK AND DELICIOUS RECIPES USING JUST FOUR INGREDIENTS OR LESS!	HUGHES, NANCY S	2016
50 HEALTHIEST HABITS AND LIFESTYLE CHANGES.	GOLDSTEIN, MYRNA CHANDLER	2016
A good life to the end : taking control of our inevitable journey through ageing and death	Ken Hillman	2017
Academy of Nutrition and Dietetics complete food and nutrition guide	Roberta Larson Duyff; Academy of Nutrition and Dietetics,	2017
ACHIEVING A HEALTHY WEIGHT FOR YOUR CHILD: AN ACTION PLAN FOR FAMILIES	HASSINK, SANDRA GIBSON	2018
ADD-FRIENDLY WAYS TO ORGANIZE YOUR LIFE: STRATEGIES THAT WORK FROM A PROFESSIONAL ORGANIZER AND A RENOWNED ADD CLINICIAN.	KOLBERG, JUDITH	2017
ADDICTED TO E-CIGARETTES AND VAPING	MOONEY, CARLA	2019
ADDICTION AND OVERDOSE: CONFRONTING AN AMERICAN CRISIS.	GOLDSMITH, CONNIE	2017
ADHD and Me: What I Learned from Lighting Fires at the Dinner Table	Taylor, Blake E. S.	2007

Title	Authors	Year published
ADHD GO-TO GUIDE: FACTS AND STRATEGIES FOR PARENTS AND TEACHERS.	SILVA, DESIREE. UNIV. OF WESTERN AUSTRALIA	2017
ADHD IN ADOLESCENTS: DEVELOPMENT, ASSESSMENT, AND TREATMENT	STEPHEN P. BECKER and UNIVERSITY OF CINCINNATI	2020
ADHD: WHAT EVERY PARENT NEEDS TO KNOW.	WOLRAICH, MARK L	2019
Adolescent Depression: A Guide for Parents (A Johns Hopkins Press Health Book)	Mondimore, Francis Mark,	2015
AFRICAN AMERICANS AND DEPRESSION: SIGNS, AWARENESS, TREATMENTS, AND INTERVENTIONS	HASTINGS, JULIA F and UNIVERSITY AT ALBANY, SUNY.	2015
AL CAPONE DOES MY SHIRTS.	CHOLDENKO, GENNIFER	2004
Algunos niños son…Four book series	Lola Schaefer	2010
ALICEHEIMER'S: ALZHEIMER'S THROUGH THE LOOKING GLASS.	WALRATH, DANA	2016
American Cancer Society complete guide to nutrition for cancer survivors: Eating well, staying well during and after cancer	Barbara Grant MS RD CSO LD (Editor), Abby S. Bloch PhD RD (Editor), Kathryn K. Hamilton MA RD CDN CSO (Editor), Cynthia A. Thomson PhD RD (Editor)	2010
American Diabetes Association complete guide to diabetes / The Ultimate Home Reference from the Diabetes Experts	American Diabetes Association.	2011
AMERICAN HEART ASSOCIATION HEALTHY SLOW COOKER COOKBOOK: 200 LOW-FUSS, GOOD-FOR-YOU RECIPES	CLARKSON POTTER	2012

Title	Authors	Year published
American Medical Association complete guide to prevention and wellness : what you need to know about preventing illness, staying healthy, and living longer	American Medical Association.	2008
ANA'S STORY: A JOURNEY OF HOPE.	BUSH, JENNA	2007
AND IN HEALTH: A GUIDE FOR COUPLES FACING CANCER TOGETHER	SHAPIRO, DAN and PENN STATE COLLEGE OF MEDICINE.	2013
ANOREXIA AND BULIMIA: DANGEROUS EATING DISORDERS.	NELSON, KRISTEN RAJCZAK	2019
ANTICANCER LIVING: TRANSFORM YOUR LIFE AND HEALTH WITH THE MIX OF SIX	COHEN, LORENZO and U Texas MD Anderson Ctr	2018
ANXIETY HAPPENS: 52 WAYS TO FIND PEACE OF MIND.	FORSYTH, JOHN P. and UNIVERSITY AT ALBANY, SUNY.	2018
ANXIETY IS REALLY STRANGE	HAINES, STEVE	2018
ARE U OK?: A GUIDE TO CARING FOR YOUR MENTAL HEALTH	MORTON, KATI	2018
ARE YOU WHAT YOU EAT?.	DORLING KINDERSLEY, INC.	2015
ASPERGER SYNDROME: THE OASIS GUIDE: ADVICE, INSPIRATION, INSIGHT, AND HOPE, FROM EARLY INTERVENTION TO ADULTHOOD.	BASHE, PATRICIA ROMANOWSKI and STONY BROOK UNIV.	2014
ASPERGERS RULES!: HOW TO MAKE SENSE OF SCHOOL AND FRIENDS.	GROSSBERG, BLYTHE	2012
AT WAR WITH YOURSELF.	WILLIAMS, SAMUEL C	2016
AT WIT'S END: PLAIN TALK ON ALZHEIMER'S FOR FAMILIES AND CLINICIANS.	KRAUS, GEORGE	2017

Title	Authors	Year published
ATTENTION-DEFICIT HYPERACTIVITY DISORDER: A HANDBOOK FOR DIAGNOSIS AND TREATMENT	RUSSELL A. BARKLEY	2018
AUTISM AND YOUR TEEN: TIPS AND STRATEGIES FOR THE JOURNEY TO ADULTHOOD.	GROSSBERG, BLYTHE N	2019
AUTISM SPECTRUM GUIDE TO SEXUALITY AND RELATIONSHIPS: UNDERSTAND YOURSELF AND MAKE CHOICES THAT ARE RIGHT FOR YOU.	GOODALL, EMMA	2016
AYUDAR A UN AMIGO CON UN PROBLEMA DE ALCOHOL/ HELPING A FRIEND WITH AN ALCOHOL PROBLEM	LANDAU, JENNIFER	2017
AYUDAR A UN AMIGO CON UN PROBLEMA DE DROGAS/ HELPING A FRIEND WITH A DRUG PROBLEM	MCKENZIE, PRECIOUS	2017
Baby Day by Day: In-Depth, Daily Advice on Your Baby s Growth, Care, and Development in the First	DORLING KINDERSLEY, INC.	2012
BABY, LET'S EAT!		2018
BEAT DEPRESSION TO STAY HEALTHIER AND LIVE LONGER: A GUIDE FOR OLDER ADULTS AND THEIR FAMILIES.	MOAK, GARY S	2016
BEBE, VAMOS A COMER!/ BABY, LET'S EAT!.	LOMP, STEPHAN	2018
BECOME YOUR CHILD'S SLEEP COACH: THE BEDTIME DOCTOR'S 5-STEP GUIDE, AGES 3-10.	SCHNEEBERG, LYNELLE and YALE SCHOOL OF MEDICINE	2019
BECOMING AN ALLY TO THE GENDER-EXPANSIVE CHILD: A GUIDE FOR PARENTS AND CARERS	BIANCHI, ANNA	2018
BEFORE AND AFTER CANCER TREATMENT: HEAL FASTER, BETTER, STRONGER.	Silver, J. K. and Harvard medical school	2015
BEFORE I FORGET: LOVE, HOPE, HELP, AND ACCEPTANCE IN OUR FIGHT AGAINST ALZHEIMErs	SMITH, B.	2016

Title	Authors	Year published
Before your time : the early menopause survival guide	Sterling, Evelina Weidman,	2010
BIG FIVE: FIVE SIMPLE THINGS YOU CAN DO TO LIVE A LONGER, HEALTHIER LIFE	CHOPRA, SANJIV and HARVARD MEDICAL SCHOOL	2016
BIRD IN A CAGE.	ROHER, REBECCA	2016
BIRTH GUY'S GO-TO GUIDE FOR NEW DADS: HOW TO SUPPORT YOUR PARTNER THROUGH BIRTH, BREASTFEEDING & BEYOND.	SALMON, BRIAN W	2019
BLACK WOMAN'S BREAST CANCER SURVIVAL GUIDE: UNDERSTANDING AND HEALING IN THE FACE OF A NATIONWIDE CRISIS.	HOLLOWAY, CHERYL D and SOUTH UNIVERSITY.	2017
BODY AND MIND: LGBTQ HEALTH ISSUES.	QUIST, JEREMY	2019
Borderline personality disorder demystified :	Friedel, Robert O.,	2018
BOUNCING BACK: SKILLS FOR ADAPTATION TO INJURY, AGING, ILLNESS, AND PAIN.	WANLASS, RICHARD and UNIVERSITY OF CALIFORNIA	2017
Brain on fire : my month of madness	Cahalan, Susannah.	2013
BREAST CANCER SURGERY AND RECONSTRUCTION: WHAT'S RIGHT FOR YOU	ANSTETT, PATRICIA	2016
BREAST TEST BOOK: A WOMAN'S GUIDE TO MAMMOGRAPHY AND BEYOND.	JONES, CONNIE and U New Mexico Schl of Med	2017
BUENA SALUD GUIDE FOR A HEALTHY HEART.	DELGADO, JANE L	2011
BUENA SALUD GUIDE TO DIABETES AND YOUR LIFE.	DELGADO, JANE L	2011
CALMING YOUR ANXIOUS CHILD: WORDS TO SAY AND THINGS TO DO	TRAINOR, KATHLEEN and Harvard	2016
CAMOUFLAGE: THE HIDDEN LIVES OF AUTISTIC WOMEN.	BARGIELA, SARAH	2019

Title	Authors	Year published
Can I tell you about ADHD? : a guide for friends, family and professionals	Susan Yarney	2013
Can I tell you about Asperger syndrome? : a guide for friends and family	Jude Welton; Jane Telford	2004
Can I tell you about cerebral palsy? : a guide for friends, family and professionals	Marion Stanton; Katie Stanton	2014
Can I tell you about dementia? : a guide for family, friends and carers	Jude Welton	2013
Can I tell you about depression? : a guide for friends, family and professionals	Christopher Dowrick; Susan Martin; Mike Medaglia	2015
Can I tell you about diabetes (type 1)? : a guide for friends, family and professionals	Julie Edge; Julia MacConville	2014
Can I tell you about dyslexia? : a guide for friends, family, and professionals	Alan M Hultquist; Bill Tulp	2013
Can I tell you about dyspraxia? : a guide for friends, family and professionals	Maureen Boon; Imogen Hallam	2014
Can I tell you about epilepsy? : a guide for friends, family and professionals	Kate Lambert; Scott Hellier	2012
Can I tell you about Parkinson's disease? : a guide for family, friends, and carers	Alan M Hultquist; Lydia T Corrow	2013
CANCER COMPANION: AN ONCOLOGIST'S ADVICE ON DIAGNOSIS, TREATMENT, AND RECOVERY.	SRIVASTAVA, RANJANA	2015
Cancer in our family : helping children cope with a parent's illness	Sue P Heiney; Joan F Hermann	2013

Title	Authors	Year published
Cancer is a word not a sentence: A practical guide to help you through the first few weeks	Dr Robert Buckman	2007
CANCER NUTRITION & RECIPES FOR DUMMIES.	MARKHAM, MAURIE	2013
CANCER PREVENTION MANUAL: SIMPLE RULES TO REDUCE THE RISKS	OLVER, IAN N and UNIVERSITY OF SOUTH AUSTRALIA	2016
CANCER VIXEN: A TRUE STORY.	MARCHETTO, MARISA ACOCELLA	2009
CANCER VIXEN: MI LUCHA CONTRA EL CANCER.	MARCHETTO, MARISA ACOCELLA	2007
CAN'T WE TALK ABOUT SOMETHING MORE PLEASANT?	CHAST, ROZ	2014
CAREGIVER'S ENCYCLOPEDIA: A COMPASSIONATE GUIDE TO CARING FOR OLDER ADULTS.	GILLICK, MURIEL R	2020
CAREGIVING IN ALZHEIMER'S AND OTHER DEMENTIAS.	PFEIFFER, ERIC and UNIV. OF SOUTH FLORIDA	2015
CARING FOR AUTISM: PRACTICAL ADVICE FROM A PARENT AND PHYSICIAN.	ELLIS, MICHAEL A and MERCER UNIVERSITY SCHOOL OF MEDICINE.	2018
CARING FOR AUTISM: PRACTICAL ADVICE FROM A PARENT AND PHYSICIAN.	Ellis, Michael A.,	2018
Caring for your baby and young child : birth to age 5		2019
CHEF RONALDO'S SABORES DE CUBA: DIABETES-FRIENDLY TRADITIONAL AND NUEVA CUBANO CUISINE.	LINARES, RONALDO	2016
CHILDREN'S BOOK OF HEALTHY EATING: IMPROVING LIVES THROUGH BETTER NUTRITION.	STIMPSON, JO	2016
CHILDREN'S MEDICINES: WHAT EVERY PARENT, GRANDPARENT, AND TEACHER NEEDS TO KNOW.	BELL, EDWARD A. and Drake University	2017

Title	Authors	Year published
CHRONIC ILLNESSES, SYNDROMES, AND RARE DISORDERS: THE ULTIMATE TEEN GUIDE.	BRILL, MARLENE TARG	2016
Cleveland clinic healthy heart lifestyle guide and cookbook	Polin, Bonnie Sanders,	2007
Clinical trials : what patients and healthy volunteers need to know	Lorna Speid	2010
Coco y Tula buenos hábitos. Eight part series.	Patricia Geis and Sergio Folch	2006
COGNITIVE BEHAVIORAL THERAPY FOR ANXIETY AND DEPRESSION DURING PREGNANCY AND BEYOND: HOW TO MANAGE SYMPTOMS AND MAXIMIZE WELL-BEING.	GREEN, SHERYL M and MCMASTER UNIVERSITY	2019
Cómo cuidar mi salud, five book series	Mari Schuh, Sarah Seurette, Terri DeGezelle	2007
Cómo mantenernos saludables. Four book series, all health related	Amanda Doering Tourville and Ronnie Rooney	2012
Cómo meditar y ser al mismo tiempo un buen amigo de tu mente	Pema Chödrön	2013

Title	Authors	Year published
COMPLETE FAMILY GUIDE TO ADDICTION: EVERYTHING YOU NEED TO KNOW NOW TO HELP YOUR LOVED ONE AND YOURSELF.	HARRISON, THOMAS F	2019
COMPLETE GUIDE TO CARB COUNTING: PRACTICAL TOOLS FOR BETTER DIABETES MEAL PLANNING	AMERICAN DIABETES ASSN	2019
COMPLETE MONTH OF MEALS COLLECTION.	AMERICAN DIABETES ASSN	2017
CONCEPTION.	BUCKHANON, KALISHA	2008
CONFRONTING CHRONIC PAIN: A PAIN DOCTOR'S GUIDE TO RELIEF.	RICHEIMER, STEVEN and UNIVERSITY OF SOUTHERN CALIFORNIA	2014
CONVERSATION TRAIN: A VISUAL APPROACH TO CONVERSATION FOR CHILDREN ON THE AUTISM SPECTRUM	SHAUL, JOEL	2014
COPING WITH BIPOLAR DISORDER.	GORDON, SHERRI MABRY	2019
COPING WITH GENDER FLUIDITY.	LUNDQUIST-ARORA, STEPHANIE	2020
COPING WITH HIV AND AIDS.	HOMPSON, ELISSA	2019
Coping with post-traumatic stress disorder : a guide for families	Roberts, Cheryl A.	2011
COPING WITH SEXUALLY TRANSMITTED DISEASES	PARRISH, JACQUELINE	2020
CREATING MOMENTS OF JOY: ALONG THE ALZHEIMER'S JOURNEY.	BRACKEY, JOLENE	2017
CRIBSHEET: A DATA-DRIVEN GUIDE TO BETTER, MORE RELAXED PARENTING, FROM BIRTH TO PRESCHOOL.	OSTER, EMILY and Brown University	2019

Title	Authors	Year published
CUP OF WATER UNDER MY BED: A MEMOIR.	HERNANDEZ, DAISY	2014
DADS, KIDS, AND FITNESS: A FATHER'S GUIDE TO FAMILY HEALTH.	MARSIGLIO, WILLIAM and University of Florida	2016
DANGERS OF ALCOHOL.	THIEL, KRISTIN	2020
DANGERS OF METHAMPHETAMINE.	MARCOVITZ, HAL	2016
DANGERS OF OPIOIDS.	KLIMCHUK, DAVID	2020
DANGERS OF PRESCRIPTION DRUGS	THIEL, KRISTIN	2020
DANGERS OF TOBACCO.	TOLLI, JENNA	2020
DANGERS OF VAPING.	IDZIKOWSKI, LISA	2020
DEAR SCARLET: THE STORY OF MY POSTPARTUM DEPRESSION.	WONG, TERESA	2019
DECOLONIZE YOUR DIET: PLANT-BASED MEXICAN-AMERICAN RECIPES FOR HEALTH AND HEALING.	CALVO, LUZ and Cal State Univ/East Bay	2015
DEMENTIA CAREGIVER: A GUIDE TO CARING FOR SOMEONE WITH ALZHEIMER'S DISEASE AND OTHER NEUROCOGNITIVE DISORDERS.	AGRONIN, MARC E and UNIVERSITY OF MIAMI MILLER SCHOOL OF MEDICINE	2016
DEPLOYMENT TOOLKIT: MILITARY FAMILIES AND SOLUTIONS FOR A SUCCESSFUL LONG-DISTANCE RELATIONSHIP.	MOORE, JANELLE	2016
DEPRESSION IN LATER LIFE: AN ESSENTIAL GUIDE.	SERANI, DEBORAH and ADELPHI UNIVERSITY	2016
DESIGNED FOR ONE!: 120 DIABETES-FRIENDLY DISHES JUST FOR YOU.	hUGHES, NANCY S	2017

Title	Authors	Year published
DETECTING & LIVING WITH BREAST CANCER FOR DUMMIES.	GEORGE, MARSHALEE and JOHNS HOPKINS UNIVERSITY	2017
DIABETES & KEEPING FIT FOR DUMMIES.	COLBERG, SHERI	2018
DIABETES 2-MONTH TURNAROUND.	AMERICAN DIABETES ASSN	2017
DIABETES A TO Z: WHAT YOU NEED TO KNOW ABOUT DIABETES - SIMPLY PUT.	AMERICAN DIABETES ASSN	2016
DIABETES AND WELLBEING: MANAGING THE PSYCHOLOGICAL AND EMOTIONAL CHALLENGES OF DIABETES TYPES 1 AND 2.	NASH, JEN	2013
DIABETES CARBOHYDRATE & FAT GRAM GUIDE: QUICK, EASY MEAL PLANNING USING CARBOHYDRATE AND FAT GRAM COUNTS.	HOLZMEISTER, LEA ANN	2017
DIABETES DE LA A A LA Z: LO QUE NECESITA SABER SOBRE LA DIABETES EN TERMINOS SIMPLES.	AMERICAN DIABETES ASSN	2016
DIABETES HEAD TO TOE: EVERYTHING YOU NEED TO KNOW ABOUT DIAGNOSIS, TREATMENT, AND LIVING WITH DIABETES.	KALYANI, RITA RASTOGI and Johns Hopkins	2018
DIABETES MEAL PLANNING MADE EASY.	WARSHAW, HOPE S.,	2016
DIABETES SUPERFOODS COOKBOOK AND MEAL PLANNER: POWER-PACKED RECIPES AND MEAL PLANS DESIGNED TO HELP YOU LOSE WEIGHT AND CONTROL YOUR BLOOD…	verdi, c	2019
DIABETES-AWARE DIETS	SCHUH, MARI	2014
DIABETES--HOW TO HELP: YOUR COMPLETE GUIDE TO CARING FOR A LOVED ONE WITH DIABETES	SCHEINER, GARY	2018
DISABILITIES, SEXUAL HEALTH, AND CONSENT.	RATCLIFF, ACE	2019
Don't panic : taking control of anxiety attacks	Wilson, Robert R.	2009
DR. PATRICK WALSH'S GUIDE TO SURVIVING PROSTATE CANCER.	walsh, p	2018

Title	Authors	Year published
DRAWN TO SEX: OUR BODIES AND HEALTH.	MOEN, ERIKA	2020
DYSLEXIA ADVOCATE!: HOW TO ADVOCATE FOR A CHILD WITH DYSLEXIA WITHIN THE PUBLIC EDUCATION SYSTEM.	SANDMAN-HURLEY, KELLI	2016
DYSLEXIA IS MY SUPERPOWER (MOST OF THE TIME).	ROOKE, MARGARET	2017
EAT HEALTHY FOODS.	MARSICO, KATIE	2019
EAT LESS SALT: AN EASY ACTION PLAN FOR FINDING AND REDUCING THE SODIUM HIDDEN IN YOUR DIET WITH 60 HEART- HEALTHY RECIPES.	CLARKSON POTTER	2013
EAT YOUR GREENS, REDS, YELLOWS, AND PURPLES.	DORLING KINDERSLEY, INC.	2016
EATING HEALTHY.	BODDEN, VALERIE	2015
EL DEAFO	BELL, CECE	2014
El deporte y mi cuerpo. Series of 5 books.	Charlotte Guillain and Catherine Veitch	2011
EL MANUAL DEL PACIENTE : C?MO SALVAR SU VIDA Y LA DE SUS SERES QUERIDOS	Leslie D Michelson	2016
EMPOWERING GUIDE TO LUNG CANCER: SIX STEPS TO TAKING CHARGE OF YOUR CARE AND YOUR LIFE.	PRESSER, ERIC	2017
END OF YOUR LIFE BOOK CLUB.	SCHWALBE, WILL	2012
ESSENTIAL GUIDE TO FAMILY & MEDICAL LEAVE	GUERIN, LISA	2018

Title	Authors	Year published
Everyone's guide to cancer therapy: How cancer is diagnosed, treated, and managed day to day.	Andrew H Ko; Malin Dollinger; Ernest H Rosenbaum	2008
EVERYTHING HAPPENS FOR A REASON: AND OTHER LIES I'VE LOVED.	BOWLER, KATE and Duke University	2018
EVERYTHING YOU NEED TO KNOW ABOUT BIRTH CONTROL.	BENSON, ALANA	2019
EVERYTHING YOU NEED TO KNOW ABOUT SMOKING, VAPING,AND YOUR HEALTH.	GORDON, SHERRI MABRY	2019
EVERYTHING YOU NEED TO KNOW ABOUT THE RISKS OF UNPROTECTED SEX.	DECARLO, CAROLYN	2019
EXPECTING MINDFULLY: NOURISH YOUR EMOTIONAL WELL-BEING AND PREVENT DEPRESSION DURING PREGNANCY AND POSTPARTUM.	DIMIDJIAN, SONA and Emory Uni	2019
EXPLORING DEPRESSION, AND BEATING THE BLUES: A CBT SELF-HELP GUIDE TO UNDERSTANDING AND COPING WITH DEPRESSION IN ASPERGER'S.	ATTWOOD, TONY and Griffith University	2016
fACTS OF LIFE.	KNIGHT, PAULA	2017
Fall down 7 times get up 8: a young man's voice from the silence of autism	Naoki Higashida	2017
FAMILY GUIDE TO MENTAL ILLNESS AND THE LAW: A PRACTICAL HANDBOOK.	TASHBOOK, LINDA	2019
FANTASTIC FRUIT RECIPES	RAJCZAK, KRISTEN	2014
FIGHTING CANCER WITH KNOWLEDGE & HOPE: A GUIDE FOR PATIENTS, FAMILIES, AND HEALTH CARE PROVIDERS.	WEILL CORNELL MEDICAL COLLEGE and FRANK, RICHARD C	2013
FINDING AUDREY	KINSELLA, SOPHIE	2015
FINDING YOUR EMOTIONAL BALANCE: A GUIDE FOR WOMEN.	MILLER, MERRY NOEL and EAST TENNESSEE STATE UNIVERSITY	2015

Title	Authors	Year published
Finishing our story : preparing for the end of life	Gregory L Eastwood	2019
FIRST YEAR OUT: A TRANSITION STORY.	SYMINGTON, SABRINA	2018
FLASH COUNT DIARY: MENOPAUSE AND THE VINDICATION OF NATURAL LIFE.	STEINKE, DARCEY	2019
FOOD & FITNESS AFTER 50: EAT WELL, MOVE WELL, BE WELL.	ROSENBLOOM, CHRISTINE	2018
FOOD ALLERGIES: THE ULTIMATE TEEN GUIDE	REINO, JESSICA	2015
FOOD FIGHT: A GRAPHIC GUIDE ADVENTURE.	O'DONNELL, LIAM	2010
FRIEND FOR HENRY.	BAILEY, JENN	2019
Full catastrophe living : using the wisdom of your body and mind to face stress, pain, and illness	Jon Kabat-Zinn; University of Massachusetts Medical Center/Worcester. Stress Reduction Clinic.	2013
GAY & BISEXUAL MEN LIVING WITH PROSTATE CANCER: FROM DIAGNOSIS TO RECOVERY	JANE M. USSHER and WESTERN SYDNEY UNIVERSITY	2018
GENDER QUEST WORKBOOK: A GUIDE FOR TEENS & YOUNG ADULTS EXPLORING GENDER IDENTITY.	TESTA, RYLAN JAY	2016
GET INTO SMART SNACKS.	DYER, JANICE	2017
GET INTO SMOOTHIES	WINTERS, JAIME	2017
GET THE MOST OUT OF RETIREMENT: CHECKLIST FOR HAPPINESS, HEALTH, PURPOSE, AND FINANCIAL	HURME, SALLY BALCH	2017
GETTING AHEAD OF ADHD: WHAT NEXT-GENERATION SCIENCE SAYS ABOUT TREATMENTS THAT WORK - AND HOW YOU CAN MAKE THEM WORK FOR YOUR CHILD.	NIGG, JOEL T and OREGON HEALTH & SCIENCE UNIVERSITY	2017

Title	Authors	Year published
GHOSTS.	TELGEMEIER, RAINA	2016
GO RED FOR WOMEN COOKBOOK: COOK YOUR WAY TO A HEART-HEALTHY WEIGHT AND GOOD NUTRITION	DEBORAH RENZA	2013
GOODBYE GLUTEN: HAPPY HEALTHY DELICIOUS EATING WITH A TEXAS TWIST	STANFORD, KIM and UNIV OF NORTH TEXAS PRESS	2014
GUIDE TO GOOD MENTAL HEALTH ON THE AUTISM SPECTRUM	PURKIS, JEANETTE	2016
GUIDE TO SURVIVORSHIP FOR WOMEN WHO HAVE OVARIAN CANCER	ROBERT E. BRISTOW and UNIV. OF CALIFORNIA, IRVINE.	2015
GUILT, SHAME, AND ANXIETY: UNDERSTANDING AND OVERCOMING NEGATIVE EMOTIONS.	BREGGIN, PETER ROGER	2014
Guts	TELGEMEIER, RAINA	2019
Hablemos del cuerpo humano, Eight book series	Cynthia Klingel and Robert B. Noyed	2010
HEADCASE: LGBTQ WRITERS AND ARTISTS ON MENTAL HEALTH AND WELLNESS	STEPHANIE SCHROEDER	2019
HEALTHY BEGINNINGS: GIVING YOUR BABY THE BEST START, FROM PRECONCEPTION TO BIRTH	JENNIFER BLAKE and UNIV. OF ALBERTA.	2017
HEALTHY EATING HABITS	REINKE, BETH BENCE	2018
HEALTHY FOODS AROUND THE WORLD	REINKE, BETH BENCE	2018

Title	Authors	Year published
HEALTHY FOODS MAKE YOUR BODY GO	MASON, DAVID I. A	2015
HEALTHY HEART BOOK.	GLASGOW CALEDONIAN UNIVERSITY AND THOW, MORAG K	2013
HELPING A FRIEND WITH A DRUG PROBLEM.	MCKENZIE, PRECIOUS	2017
HELPING A FRIEND WITH AN ALCOHOL PROBLEM.	LANDAU, JENNIFER	2017
HELPING TEENS WHO CUT: USING DBT SKILLS TO END SELF-INJURY	HOLLANDER, MICHAEL and HARVARD MEDICAL SCHOOL	2017
Helping your anxious teen :	Josephs, Sheila Achar,	2017
HELPING YOUR ANXIOUS TEEN: POSITIVE PARENTING STRATEGIES TO HELP YOUR TEEN BEAT ANXIETY, STRESS, AND WORRY. Author:JOSEPHS, SHEILA ACHAR	JOSEPHS, SHEILA ACHAR	2017
HELPING YOUR CHILD WITH LANGUAGE-BASED LEARNING DISABILITIES: STRATEGIES TO SUCCEED IN SCHOOL & LIFE WITH DYSLEXIA, DYSGRAPHIA, DYSCALCULIA	FRANKLIN, DANIEL	2018
Hey, Kiddo	KROSOCZKA, JARRETT J	2018
How to be a patient : the essential guide to navigating the world of modern medicine	Sana Goldberg	2019
HOW TO CHOOSE FOODS YOUR BODY WILL USE.	SJONGER, REBECCA	2016
HOW TO UNDERSTAND YOUR GENDER: A PRACTICAL GUIDE FOR EXPLORING WHO YOU ARE.	IANTAFFI, ALEX	2018
HPV: PREVENTION AND TREATMENT.	HARRIS, MICHELLE	2018
I EAT WELL.	RUSTAD, MARTHA E. H	2017

Title	Authors	Year published
IF YOUR ADOLESCENT HAS ADHD: AN ESSENTIAL RESOURCE FOR PARENTS.	POWER, THOMAS J	2018
I'M ALLERGIC TO MILK.	NELSON, MARIA	2014
I'M ALLERGIC TO PEANUTS.	NELSON, MARIA	2014
IN CASE YOU'RE CURIOUS: WHAT YOUNG PEOPLE ARE ASKING ABOUT SEX WITH ANSWERS FROM THE EXPERTS.	PLANNED PARENTHOOD	2019
INDIAN CUISINE DIABETES COOKBOOK: SAVORY SPICES AND BOLD FLAVORS FROM SOUTH ASIA.	FRIDEL, MAY ABRAHAM	2017
INFORMED PATIENT: A COMPLETE GUIDE TO A HOSPITAL STAY.	FRIEDMAN, KAREN A and HOFSTRA NORTHWELL SCHOOL OF MEDICINE	2017
INSULIN PUMPS AND CONTINUOUS GLUCOSE MONITORING: A USER'S GUIDE TO EFFECTIVE DIABETES MANAGEMENT.	KAUFMAN, FRANCINE RATNER and UNIVERSITY OF SOUTHERN CALIFORNIA	2017
IT'S PERFECTLY NORMAL: CHANGING BODIES, GROWING UP, SEX, AND SEXUAL HEALTH.	HARRIS, ROBIE H	2014
JOHN HOPKINS PATIENTS GUIDE TO COLON AND RECTAL CANCER	AHUJA, NITA and JOHNS HOPKINS UNIV SCHOOL OF MEDICINE	2014
Just move! : a new approach to fitness after 50	James P. Owen with Brigitte LeBlanc	2017
Keeping your kids out of the emergency room : a guide to childhood injuries and illnesses	Christopher M Johnson	2014
KEYS TO PARENTING YOUR ANXIOUS CHILD.	MANASSIS, KATHARINA and UNIVERSITY OF TORONTO	2015

Title	Authors	Year published
KIDS, MUSIC 'N' AUTISM: BRINGING OUT THE MUSIC IN YOUR CHILD.	BERGER, DORITA S	2017
LATIN COMFORT FOODS MADE HEALTHY: MORE THAN 100 DIABETES-FRIENDLY LATIN FAVORITES = CLASICOS LATINOS A LO SALUDABLE.	HOFFMANN, INGRID	2018
LATINA GUIDE TO HEALTH: CONSEJOS AND CARING ANSWERS.	DELGADO, JANE L	2010
LENA'S SHOES ARE NERVOUS: A FIRST-DAY-OF-SCHOOL DILEMMA	CALABRESE, KEITH	2018
LIGHTER THAN MY SHADOW	GREEN, KATIE	2017
Limpieza y salud (todo el día), 5 book series	Elizabeth Vogel	2003 - 2006
LIVING A HEALTHY LIFESTYLE.	ALLMAN, TONEY	2019
LIVING LONGER, LIVING BETTER: LIFESTYLE, EXERCISE, DIET, AND YOGA FOR HEART AND MIND.	OPIE, LIONEL H	2016
LIVING PROUD! KEEPING PHYSICALLY HEALTHY.	RODI, ROBERT	2016
Living safely, aging well : a guide to preventing injuries at home	Dorothy A Drago; Johns Hopkins University Press.	2013
LIVING WITH ADHD	HORNING, NICOLE	2018

Title	Authors	Year published
LIVING WITH ALLERGIES.	BURKHART, JULIANA	2019
LIVING WITH CANCER: A STEP-BY-STEP GUIDE FOR COPING MEDICALLY AND EMOTIONALLY WITH A SERIOUS DIAGNOSIS.	JACKSON, VICKI A	2017
LONG ROAD HOME: ONE STEP AT A TIME.	TRUDEAU, GARY B	2005
LONGEVITY PLAN: SEVEN LIFE-TRANSFORMING LESSONS FROM ANCIENT CHINA.	DAY, JOHN D	2017
LONG-TERM CARE: HOW TO PLAN AND PAY FOR IT	MATTHEWS, J. L.,	2018
Los buenos hábitos en pop-up	Patricia Geis	2011 and 2014
LOVING APPROACH TO DEMENTIA CARE: MAKING MEANINGFUL CONNECTIONS WITH THE PERSON WHO HAS ALZHEIMER'S DISEASE OR OTHER DEMENTIA OR MEMORY LOSS.	WAYMAN, LAURA,	2017
LOVING, SUPPORTING, AND CARING FOR THE CANCER PATIENT: A GUIDE TO COMMUNICATION, COMPASSION, AND COURAGE.	GOLDBERG, STAN	2016
LUNG CANCER: YOUR QUESTIONS, EXPERT ANSWERS.	SCHILLER, J.H. and VIRGINIA COMMONWEALTH UNIVERSITY	2017
MAKING TOUGH DECISIONS ABOUT END-OF-LIFE CARE IN DEMENTIA.	KENNY, ANNE and UNIVERSITY OF CONNECTICUT.	2018
MANAGING HOT FLUSHES AND NIGHT SWEATS: A COGNITIVE BEHAVIOURAL SELF-HELP GUIDE TO THE MENOPAUSE.	HUNTER, MYRA and KING'S COLLEGE LONDON.	2014
MANAGING PROSTATE CANCER: A GUIDE FOR LIVING BETTER.	ROTH, ANDREW J	2015
mANAGING YOUR CHILD'S CHRONIC PAIN	ALERMO, TONYA M and UNIV. OF WASHINGTON SCHOOL OF MEDICINE.	2015
MANAGING YOUR DEPRESSION: WHAT YOU CAN DO TO FEEL BETTER.	NOONAN, SUSAN J.	2013

Title	Authors	Year published
Man's Guide to Healthy Aging : Stay Smart, Strong, and Active.	Edward H Jr Thompson	2013
MARBLES: MANIA, DEPRESSION, MICHELANGELO, + ME.	FORNEY, ELLEN	2012
MARRIAGE AND HEALTH: THE WELL-BEING OF SAME-SEX COUPLES	HUI LIU and MICHIGAN STATE UNIVERSITY.	2020
MARVIN'S MONSTER DIARY: TROUBLE WITH FRIENDS BUT I GET BY, BIG TIME! AN ST4 MINDFULNESS BOOK FOR KID S	MELMED, RAUN	2020
Maybe you should talk to someone : a therapist, her therapist, and our lives revealed	Lori Gottlieb	2019
Mayo Clinc Guide to Raising a Healthy Child	Angela C Mattke	2019
Mayo Clinic Guide to a Health Pregnancy	Myra Wick	2018
ME AND MY FEAR.	SANNA, FRANCESCA	2018
MEDICAL BILLING & CODING FOR DUMMIES.	SMILEY, KAREN	2020
MEDICARE FOR DUMMIES.	BARRY, PATRICIA	2018
MEMORY AND COMMUNICATION AIDS FOR PEOPLE WITH DEMENTIA	BOURGEOIS, MICHELLE S. and U SOUTH FLORIDA. GUIDEBOOK	2014
Menopause Confidential: A Doctor Reveals the Secrets to Thriving Through Midlife	Allman, Tara,	2017
MENOPAUSE MAZE: THE COMPLETE GUIDE TO CONVENTIONAL, COMPLEMENTARY AND SELF-HELP OPTIONS.	ARROLL, MEGAN A	2016
MENOPAUSE: A COMIC TREATMENT	MK CZERWIEC	2020
MES DE COMIDAS: SABOR FESTIVO LATINO=MONTH OF MEALS: FESTIVE LATIN FLAVOR.	AMER DIABETES ASSN	2003
Mi amigo es, four book series	Amanda Doering Tourville and Christine Sorra	2012

Title	Authors	Year published
MINDFUL GUIDE TO MANAGING DIABETES: YOUR PATH TO REDUCING STRESS AND LIVING WELL.	NAPORA, JOSEPH	2019
MINDFUL TWENTY-SOMETHING: LIFE SKILLS TO HANDLE STRESS.	ROGERS, HOLLY and Duke University	2016
MiPlato series, seven book series	Mari Schuh	2013
MOM'S CANCER.	FIES, BRIAN	2006
MR. FOOD TEST KITCHEN GUILT-FREE COMFORT FAVORITES: MORE THAN 130 NEW HEALTHY AND DIABETES-FRIENDLY RECIPES.	AMERICAN DIABETES ASSN	2018
NATURALLY HEALTHY MEXICAN COOKING: AUTHENTIC RECIPES FOR DIETERS, DIABETICS, & ALL FOOD LOVERS.	PEYTON, JAMES W	2014
Navigating breast cancer : a guide for the newly diagnosed	Lillie Shockney	2011
NAVIGATING THE COMPLEXITIES OF STROKE.	CAPLAN, LOUIS R AND HARVARD UNIVERSITY. CO-PUB. WITH AMERICAN ACADEMY OF NEUROLOGY.	2013
NEW AMERICAN HEART ASSOCIATION COOKBOOK	HARMONY CROWN	2017
NEW SOUL FOOD COOKBOOK FOR PEOPLE WITH DIABETES	GAINES, FABIOLA	2018
NOT SO DIFFERENT: WHAT YOU REALLY WANT TO ASK ABOUT HAVING A DISABILITY.	BURCAW, SHANE	2017
Nuestros asombroso cuerpo: los cincos sentidos, Five book series	Dana Meachen Rau and Rick Peterson	2008

Title	Authors	Year published
Nutrition : what every parent needs to know	William H Dietz; Loraine Stern; American Academy of Pediatrics.	2012
OBESITY EPIDEMIC: WHY DIETS AND EXERCISE DON'T WORK--AND WHAT DOES.	TOOMATH, ROBYN	2017
OBSESSED: A MEMOIR OF MY LIFE WITH OCD	BRITZ, ALLISON	2017
ON THE COME UP: A NOVEL, BASED ON A TRUE STORY	WEYER, HANNAH	2013
ONCE A WARRIOR ALWAYS A WARRIOR: NAVIGATING THE TRANSITION FROM COMBAT TO HOME- INCLUDING COMBAT STRESS, PTSD	HOGE, CHARLES W	2010
OPIOIDS: HEROIN, OXYCONTIN, AND PAINKILLERS	PERRITANO, JOHN	2016
OUR FOOD: A HEALTHY SERVING OF SCIENCE AND POEMS	LIN, GRACE	2018
PAIN IS REALLY STRANGE	HAINES, STEVE	2015
PARENTING CHILDREN WITH ADHD: 10 LESSONS THAT MEDICINE CANNOT TEACH	MONASTRA, VINCENT J	2014
Patient safety survival guide : how to protect yourself and others from medical errors	Gretchen LeFever Watson	2017
PATIENTS' GUIDE TO CANCER IN OLDER ADULTS	GARY R. SHAPIRO and JOHNS HOPKINS.	2012
PEANUT AND OTHER FOOD ALLERGIES.	MCANENEY, CAITIE	2015
PERFECT DIABETES COMFORT FOOD COLLECTION: 9 ESSENTIAL RECIPES YOU NEED TO CREATE 90 AMAZING COMPLETE MEALS.	WEBB, ROBYN	2016
PERFORMANCE-ENHANCING DRUGS: STEROIDS, HORMONES, AND SUPPLEMENTS.	PERRITANO, JOHN	2016
PICKY EATER PROJECT: 6 WEEKS TO HAPPIER, HEALTHIER FAMILY MEALTIMES.	MUTH, NATALIE DIGATE	2017
Pilates for living : get stronger, fitter and healthier for an active later life	Angell, Harri,	2018

Title	Authors	Year published
Positive Parenting for Bipolar Kids	McDonnell, Mary Ann.	2009
PREDIABETES: A COMPLETE GUIDE: YOUR LIFESTYLE RESET TO STOP PREDIABETES AND OTHER CHRONIC ILLNESSES.	WEISENBERGER, JILL	2018
PREGNANCY AND PARENTING: THE ULTIMATE TEEN GUIDE.	AKIN, JESSICA	2016
PREGNANCY INFORMATION FOR TEENS: HEALTH TIPS ABOUT TEEN PREGNANCY AND TEEN PARENTING: INCLUDING FACTS ABOUT PRENATAL CARE, PREGNANCY COMPLICATIONS, LABOR AND DELIVERY,.	OMNIGRAPHICS	2017
PREVENTING AND REVERSING HEART DISEASE FOR DUMMIES	RIPPE, JAMES M and UNIV. OF CENTRAL FLORIDA	2015
Preventing medication errors at home	Simon Haroutounian	2020
PRIDE GUIDE: A GUIDE TO SEXUAL AND SOCIAL HEALTH FOR LGBTQ YOUTH	LANGFORD, JO	2018
PRINCESS AND THE FOG: A STORY FOR CHILDREN WITH DEPRESSION	JONES, LLOYD	2015
PSYCHIATRIC TALES: ELEVEN GRAPHIC STORIES ABOUT MENTAL ILLNESS	CUNNINGHAM, DARRYL	2011
PUTTING ON THE BRAKES: UNDERSTANDING AND TAKING CONTROL OF YOUR ADD OR ADHD	QUINN, PATRICIA O	2012
QIGONG FOR WELLBEING IN DEMENTIA AND AGING	RATH, STEPHEN	2016
Qué hacer cuando su niño se enferma? : fácil de leer, fácil de usar	Gloria G Mayer; Ann Kuklierus	2012
Qué hacer para la salud de las personas mayores : fácil de leer, fácil de usar	Albert E Barnett; Nancy Rushton	2013

Title	Authors	Year published
Qué hacer para la salud de los adolescentes : fácil de leer, fácil de usar	Gloria G Mayer; Ann Kuklierus	2007
Qué hacer para los niños con sobrepeso : fácil de leer, fácil de usar	Gloria G Mayer; Michael Villaire	2012
Que hacer para tener dientes sanos : fácil de leer, fácil de usar	Sadie S Mestman; Ariella D Herman	2011
QUICK & EASY COOKBOOK: MORE THAN 200 HEALTHY RECIPES YOU CAN MAKE IN MINUTES	American heart assoc	2012
QUICK & EASY GUIDE TO QUEER & TRANS IDENTITIES.	G., MADY	2019
QUICK & EASY GUIDE TO SEX & DISABILITY.	ANDREWS, A	2020
Raising Cubby : a father and son's adventures with Asperger's, trains, tractors, and high explosives	Robison, John Elder.	2014
RECOVERING OUR ANCESTORS' GARDENS: INDIGENOUS RECIPES AND GUIDE TO DIET AND FITNESS.	MIHESUAH, DEVON A and University of Kansas	2005
RELATIVELY INDOLENT BUT RELENTLESS: A CANCER TREATMENT JOURNAL	FREEDMAN, MATT and UNIV. OF PENNSYLVANIA SCHOOL OF DESIGN	2014
RELIEF FROM HOT FLASHES: THE NATURAL, DRUG-FREE PROGRAM TO REDUCE HOT FLASHES, IMPROVE SLEEP, AND EASE STRESS.	ELKINS, GARY RAY and Baylor University	2014
RX: A GRAPHIC MEMOIR.	LINDSAY, RACHEL	2018
SARAH KEY'S BACK SUFFERERS' BIBLE	KEY, SARAH and UNIVERSITY OF SALFORD	2016
SEDUCTIVE DELUSIONS: HOW EVERYDAY PEOPLE CATCH STIS.	GRIMES, JILL and UNIV. OF TEXAS	2016

Title	Authors	Year published
SEX AFTER SERVICE: A GUIDE FOR MILITARY SERVICE MEMBERS, VETERANS, AND THE PEOPLE WHO LOVE THEM.	HELMER, DREW A. and Rutgers University	2015
SEX AND CANCER: INTIMACY, ROMANCE, AND LOVE AFTER DIAGNOSIS AND TREATMENT.	UNIVERSITY OF COLORADO SCHOOL OF MEDICINE	2017
SEXUAL HEALTH INFORMATION FOR TEENS: HEALTH TIPS ABOUT SEXUAL DEVELOPMENT, REPRODUCTION, CONTRACEPT ION, AND SEXUALLY TRANSMITTED INFECTIONS	OMNIGRAPHICS	2016
SEXUALLY TRANSMITTED INFECTIONS: THE FACTS.	BARLOW, DAVID and London University	2011
SHOOK ONE: ANXIETY PLAYING TRICKS ON ME.	CHARLAMAGNE THA GOD	2019
SHOW ME ALL YOUR SCARS: TRUE STORIES OF LIVING WITH MENTAL ILLNESS	LEE GUTKIND and ARIZONA STATE UNIVERSITY	2016
SICK KIDS IN LOVE.	MOSKOWITZ, HANNAH	2019
SIGNATURE WOUND: ROCKING TBI.	TRUDEAU, G.B	2010
SKIN CANCER: AMERICAS MOST COMMON CANCER.	KURCH, PETER	2019
SKIN CONDITIONS: FROM ACNE TO ECZEMA.	REYNOLDS, DONNA	2019
SMILE.	TELGEMEIER, RAINA	2010
SNACKS	PARKER, VIC	2014
SOCIAL SECURITY, MEDICARE AND GOVERNMENT PENSIONS: GET THE MOST OUR OF YOUR RETIREMENT & MEDICAL BENEFITS.	MATTHEWS, J.L.	2019
SPECIAL EXITS	FARMER, JOYCE	2014
SPECTRUM WOMEN: WALKING TO THE BEAT OF AUTISM	BARB COOK	2018

Title	Authors	Year published
STELLA DIAZ HAS SOMETHING TO SAY.	DOMINGUEZ, ANGELA	2018
STIMULANTS: METH, COCAINE, AND AMPHETAMINES.	PERRITANO, JOHN	2016
STRAIGHT TALK ABOUT PSYCHIATRIC MEDICATIONS FOR KIDS.	WILENS, TIMOTHY E and HARVARD MEDICAL SCHOOL	2016
TAKE CONTROL OF YOUR DEPRESSION: STRATEGIES TO HELP YOU FEEL BETTER NOW.	NOONAN, SUSAN J.	2018
Taking charge of ADHD :	Barkley, Russell A.,	2013
TAKING CONTROL OF ANXIETY: SMALL STEPS FOR GETTING THE BEST OF WORRY, STRESS, AND FEAR.	MOORE, BRET A	2014
Talking to your doctor : a patient's guide to communication in the exam room and beyond	Zackary Berger	2015
TANGLES: A STORY ABOUT ALZHEIMER'S, MY MOTHER, AND ME.	LEAVITT, SARAH	2010
TEACHING LIFE SKILLS TO CHILDREN AND TEENS WITH ADHD: A GUIDE FOR PARENTS AND COUNSELORS	MONASTRA, VINCENT J	2016
TEENS AND DISTRACTED DRIVING	SIMMS, JENNIFER	2018
TEENS AND VAPING.	ALLEN, JOHN	2019
TEX-MEX DIABETES COOKING: MORE THAN 140 AUTHENTIC SOUTHWESTERN FAVORITES.	COFFEEN, KELLEY	2018
The art of dying well :	Butler, Katy,	2015
The autistic brain :	Grandin, Temple.	2013

Title	Authors	Year published
The definitive guide to thriving after cancer : a five-step integrative plan to reduce the risk of recurrence and build lifelong health	Lise Alschuler; Karolyn A Gazella	2013
The family guide to mental health care	Lloyd I Sederer	2015
The Merck manual go-to home guide for symptoms	Robert S Porter; Justin L Kaplan; Merck manual of patient symptoms.	2013
The Merck manual home health handbook	Robert S Porter; Justin L Kaplan; Barbara P Homeier; Richard K Albert	2011
The noonday demon : an anatomy of depression	Andrew Solomon	2016
The Nursing Mother's Companion, 7th Edition, with New Illustrations: The Breastfeeding Book Mothers Trust, from Pregnancy Through Weaning	Kathleen Huggins	2017
The patient's playbook : how to save your life and the lives of those you love	Leslie D Michelson	2015
The Wahls protocol :	Wahls, Terry L.	2017
THINGS TO DO IN A RETIREMENT HOME TRAILER PARK... WHEN YOU'RE 29 AND UNEMPLOYED.	WRIGHT, NYE	2015
THIS IS HOW IT ALWAYS IS.	FRANKEL, LAURIE	2017
THIS SHOULD NOT BE HAPPENING: YOUNG ADULTS WITH CANCER.	KATZ, ANNE	2014
TYPE 1 DIABETES SELF-CARE MANUAL.	WOOD, JAMIE and CASE WESTERN RESERVE UNIVERSITY SCHOOL OF MEDICINE.	2018

Title	Authors	Year published
UNDERSTANDING COMBAT RELATED POST TRAUMATIC STRESS DISORDER	MCDERMOTT, WALTER F and University of North Florida	2012
UNDERSTANDING LUNG CANCER: AN INTRODUCTION FOR PATIENTS AND CAREGIVERS.	ALI, NAHEED	2014
UNDERSTANDING WEIGHT CONTROL: MIND AND BODY STRATEGIES FOR LIFELONG SUCCESS.	SALTMAN, DEBORAH C and university of sydney and Harvard	2018
UNEXPECTED JOURNEY OF CARING: THE TRANSFORMATION FROM LOVED ONE TO CAREGIVER.	THOMSON, DONNA	2019
UNINTENDED JOURNEY: A CAREGIVER'S GUIDE TO DEMENTIA.	SHAGAM, JANET YAGODA	2013
UNIQUELY HUMAN: A DIFFERENT WAY OF SEEING AUTISM.	PRIZANT, BARRY M AND BROWN UNIVERSITY.	2015
UPWARD SPIRAL: USING NEUROSCIENCE TO REVERSE THE COURSE OF DEPRESSION, ONE SMALL CHANGE AT A TIME.	KORB, ALEX and UCLA	2015
VACCINATION INVESTIGATION: THE HISTORY AND SCIENCE OF VACCINES.	HAELLE, TARA	2018
Vaccines & your child : separating fact from fiction	Paul A Offit; Charlotte A Moser	2011
VERY TASTY VEGETABLE RECIPES.	RAJCZAK, KRISTEN	2014
WAITING FOR CANCER TO COME: WOMEN'S EXPERIENCES WITH GENETIC TESTING AND MEDICAL DECISION MAKING FOR BREAST AND OVARIAN CANCER.	HESSE-BIBER, SHARLENE NAGY and BOSTON COLLEGE	2014
WANDERING GENE AND THE INDIAN PRINCESS: RACE, RELIGION, AND DNA	WHEELWRIGHT, JEFF	2012
WAR WITHIN: ONE MORE STEP AT A TIME: A DOONESBURY BOOK.	TRUDEAU, GARY B	2006

Title	Authors	Year published
WE BUY FOOD AT THE STORE	COLL-SECK, JAYDEN	2016
WE LIKE TO EAT WELL	APRIL, ELYSE	2013
WHAT DO I EAT NOW?: A STEP-BY-STEP GUIDE TO EATING RIGHT WITH TYPE 2 DIABETES.	ROSS, TAMI	2015
WHAT IS IN YOUR LUNCH BOX?	MINDEN, CECILIA	2018
What to do for healthy teeth : easy to read, easy to use	Sadie S Mestman; Ariella D Herman	2016
What to do for senior health : easy to read, easy to use	Albert E Barnett; Nancy Rushton	2015
What to do for your teen's health : easy to read, easy to use	Gloria G Mayer; Ann Kuklierus	2013
What to do when your child gets sick	Gloria G Mayer; Ann Kuklierus	2015
What to do when your child has asthma : easy to read, easy to use	Stanley P Galant; Olga Guijon	2017
What to do when your child is heavy : easy to read, easy to use	Gloria G Mayer; Michael Villaire	2015
What to do when you're having a baby : easy to read - easy to use	Gloria G Mayer; Ann Kuklierus	2017
What to expect before you're expecting	Murkoff, Heidi Eisenberg.	2017
What to expect the first year	Eisenberg, Arlene.	2014
What to expect the second year :	Murkoff, Heidi Eisenberg.	2011

Title	Authors	Year published
What to expect the toddler years	Eisenberg, Arlene.	2009
WHAT TO FEED YOUR BABY & TODDLER: A MONTH-BY-MONTH GUIDE TO SUPPORT YOUR CHILD'S HEALTH & DEVELOPMENT.	AVENA, NICOLE M.,	2018
WHAT'S SO YUMMY?: ALL ABOUT EATING WELL AND FEELING GOOD.	HARRIS, ROBIE	2014
WHEN AN ADULT YOU LOVE HAS ADHD: PROFESSIONAL ADVICE FOR PARENTS, PARTNERS, AND SIBLINGS.	BARKLEY, RUSSELL A and MEDICAL UNIVERSITY OF SOUTH CAROLINA	2017
WHEN MY HEART JOINS THE THOUSAND	sTEIGER, AJ	2018
When someone you know has dementia :	Andrews, June,	2016
WHEN SOMEONE YOU KNOW HAS DEPRESSION: WORDS TO SAY AND THINGS TO DO.	NOONAN, SUSAN J	2016
WHEN SOMEONE YOU KNOW IS LIVING IN A DEMENTIA CARE COMMUNITY: WORDS TO SAY AND THINGS TO DO.	WONDERLIN, RACHAEL	2016
When someone you love is depressed :	Rosen, Laura Epstein.	1997
WHEN YOUNG PEOPLE WITH INTELLECTUAL DISABILITIES AND AUTISM HIT PUBERTY: A PARENTS' Q&A GUIDE TO HEALTH, SEXUALITY AND RELATIONSHIPS	JACKSON BROWN, FREDDY	2016
WHEN YOUR CHILD HURTS: EFFECTIVE STRATEGIES TO INCREASE COMFORT, REDUCE STRESS, AND BREAK THE CYCLE OF CHRONIC PAIN.	COAKLEY, RACHAEL and Harvard	2016
WHO ARE YOU?: THE KID'S GUIDE TO GENDER IDENTITY.	PESSIN-WHEDBEE, BROOK	2017
WHY DO I HAVE TO EAT MY GREENS?: BIG ISSUES FOR LITTLE PEOPLE AROUND HEALTH AND WELL-BEING.	MCCURRY, CHRIS	2016
WHY WE EAT FRUITS.	REINKE, BETH BENCE	2018
WHY WE EAT GRAINS.	REINKE, BETH BENCE	2018
WHY WE EAT PROTEIN.	REINKE, BETH BENCE	2018

Title	Authors	Year published
WHY WE EAT VEGETABLES.	REINKE, BETH BENCE	2018
WILL PUBERTY LAST MY WHOLE LIFE?: REAL ANSWERS TO REAL QUESTIONS FROM PRETEENS ABOUT BODY CHANGES... , SEX, AND OTHER GROWING-UP STUFF	METZGER, JULIE	2018
WILSON TRIES NEW FOODS	STEWART, ASHLEY	2015
WINNING STRATEGIES FOR SUCCESSFUL AGING.	PFEIFFER, ERIC and UNIVERSITY OF SOUTH FLORIDA COLLEGE OF MEDICINE	2013
WOMAN'S GUIDE TO LIVING WITH HEART DISEASE.	THOMAS, CAROLYN	2017
WOUNDED WARRIOR HANDBOOK: A RESOURCE GUIDE FOR RETURNING VETERANS: SPECIAL EDITION.	MOORE, JANELLE	2015
YOU ARE ENOUGH.	PETRO-ROY, JEN	2019
YOU WOULDN'T WANT TO LIVE WITHOUT CLEAN WATER.	CANAVAN, ROGER	2014
YOUR CHILD WITH INFLAMMATORY BOWEL DISEASE: A FAMILY GUIDE FOR CAREGIVING	MARIA OLIVA-HEMKER	2017
YOUR TYPE 2 DIABETES ACTION PLAN: TIPS, TECHNIQUES, AND PRACTICAL ADVICE FOR LIVING WELL WITH DIABETES.	american diabetes assn	2015

ISBN	Publisher	Price USD	Link	Topic/Audience	Age range
9781785924385	JESSICA KINGSLEY	$19.95		parent guide to child autism	adult
9780062896018	U North Carolina	$22.99		Stress management	adult
9781616207816	ALGONQUIN OF CHAPEL HILL	$16.95		teen mental health	young adult
	La Habra: Institute for Healthcare Advancement.			parenting in terms of children's health issues	adults
	Marshall Cavendish Benchmark			Individual organs	K–Grade 3
9780763786045	JONES & BARTLETT LEARNING	$19.95		chronic pain	adult
9781284124811	JONES & BARTLETT LEARNING	$19.95		STDs, HIV, Aids	adult
9781284164961	JONES & BARTLETT LEARNING	$19.95		Kidney cancer	adult
9781284172195	JONES & BARTLETT LEARNING	$19.95		Liver cancer	adult
9781449687571	JONES & BARTLETT LEARNING	$21.95		Lung cancer	adult
9781284090284	JONES & BARTLETT LEARNING	$19.95		ovarian cancer	adult
9781284152340	JONES & BARTLETT LEARNING	$19.95		prostate cancer	adult
9780763784553	JONES & BARTLETT	$23.95		parent guide to child diabetes	adult

ISBN	Publisher	Price USD	Link	Topic/Audience	Age range
9781580406765	AMERICAN DIABETES ASSN	$19.95		cookbook diabetes	adult
978-1580403207	AMER DIABETES ASSN	18.95		Cookbook, diabetes, healthy meals	Adult
9781580406147	AMERICAN DIABETES ASSN	$12.95		diabetes, weight loss surgery	adult
9781580405409	AMERICAN DIABETES ASSN	$12.95		heart disease, diabetes	adult
9781421422220	JOHNS HOPKINS UNIV PRESS	$55.00		caregiver, alzheimers, dementia	adult
9781580406376	AMERICAN DIABETES ASSN	$18.95		cookbook diabetes	adult
9781440834714	GREENWOOD	$58.00		teen nutrition and health behavior	young adult
9781760294816	Allen & Unwin	$15.94	http://www.worldcat.org/oclc/982431130	aging, palliative care	adults
9780544520585	Houghton Mifflin Harcourt	$14.99	http://www.worldcat.org/oclc/932050527	nutrition	adults
9781610021548	AMER ACAD OF PEDIATRICS	$16.95		nutrition, obesity, pediatrics, parent guide	adult
9781138190740	ROUTLEDGE	$29.95		Attention deficit disorder	adult
9781682825679	REFERENCEPOINT PR INC	$30.95		Tobacco, smoking cessation, teens	young adult
9781512409536	TWENTY-FIRST CENTURY	$37.32		tobacco, drugs, alcohol, teens	young adult
978-1572245228	NEW HARBINGER PUB	19.95		ADHD, Mental Health	Adolescent

ISBN	Publisher	Price USD	Link	Topic/Audience	Age range
9781742589480	UWA PUBLISHING	$29.99		Attention deficit disorder, parent guide for children	adult
9781462541836	GUILFORD	$40.00		attention deficit disorder, parent guide for teens	adult
9781610022644	AMER ACAD OF PEDIATRICS	$16.95		Attention deficit disorder, parent guide for children	adult
978-1421417905	JOHNS HOPKINS UNIV PRESS	21.95		Depression, Adolescent health	Adult
9781442230316	ROWMAN & LITTLEFIELD	$40.00		depression, African American population	adult
9780399238611	PUTNAM PUBLISHING GROUP	$17.99		fiction, child caring for sibling with Autism	ages 8-12
	capstone press			Disabilities in children and accessibility	K–Grade 2
9780271074689	PENN STATE UNIV PRESS	$19.95		personal narrative, alzheimers, graphic medicine	adult
9780944235782	American Cancer Society	$15.10	http://www.worldcat.org/oclc/1031649659	cancer	adults
9781580403306	American Diabetes Association.	$20.66	http://www.worldcat.org/oclc/1102208660	chronic illness, diabetes	adults
9780307888020	CLARKSON POTTER	$17.99		cookbook, heart disease, nutrition	adult

ISBN	Publisher	Price USD	Link	Topic/Audience	Age range
9780470251300	Wiley	$21.40	http://www.worldcat.org/oclc/951347563	wellness	adults
9780061379086	HARPERCOLLINS	$18.99		personal narrative, Latinx population, AIDs, adolescent, STDs	young adult
9781611800173	TRUMPETER BOOKS	$16.95		caregiving, cancer	adult
9781534567498	LUCENT BOOKS	$19.99		eating disorders, teens, body image	young adult
9780735220416	VIKING	$28.00		cancer prevention, healthy living	adult
9781684031108	NEW HARBINGER PUB	$14.95		anxiety	adult
9781848193895	JESSICA KINGSLEY	$12.95		anxiety, graphic novel.	adult
9780738234991	DA CAPO	$20.00		mental health	adult
9781465429445	DK PUBLISHING	$16.99		nutrition for children	child
9780385344654	HARMONY CROWN	$17.00		aspergers, autism, guide for parents for children to adulthood	adult
9781433811272	AMERICAN PSYCHOLOGICAL AS	$9.95		aspergers, autism, guide for children on social relationships and school	child and young adult
9781848192959	jessica kingsley	$12.95		veterans, PTSD, graphic novel	adults
9781557537676	PURDUE UNIVERSITY PRESS	$25.00		alzheimers, caregiving	adult

ISBN	Publisher	Price USD	Link	Topic/Audience	Age range
9781462538874	GUILFORD	$55.00		attention deficit disorder	adult
9781433830150	AMER PSYCHOLOGICAL ASSN	$19.99		autism, teens, parenting guide	adult
9781849057059	JESSICA KINGSLEY	$24.95		autism, teens, sexual health	young adult
9781499466201	ROSEN YOUNG ADULT	$34.45		alcohol, teens	young adult
9781499466140	ROSEN YOUNG ADULT	$12.95		drug use, teens	young adult
9780756689858	DK PUBLISHING	40		FAMILY & RELATIONSHIPS / PARENTING / CHILD REARING/HEALTH & FITNESS	Adult
9781523502073	WORKMAN PUBLISHING	$5.95		nutrition, child	children
9781442246614	ROWMAN & LITTLEFIELD	$40.00		depression, active aging	older adults
9781523503186	WORKMAN PUBLISHING	$5.95		nutrition, child	children
9780738285566	DA CAPO	$16.99		sleep disorders, parenting guide for children 3-10	adult
9781785920516	JESSICA KINGSLEY	$19.95		Transgender children, LGBTQ+ children, parenting guide	adult
9781421417783	JOHNS HOPKINS UNIV PRESS	18.95		Health/ Cancer	Adult
9780553447125	HARMONY CROWN	$25.00		alzheimers, personal narrative, African American population	adult

ISBN	Publisher	Price USD	Link	Topic/Audience	Age range
9781439108451	SIMON & SCHUSTER	18.99		Women's Health/ Menopause	Adult
9781250065339	ST MARTIN'S PRESS	$24.99		nutrition	adult
9781772620054	CONUNDRUM PRESS	$12.00		aging, graphic medicine	adults, older adults
9781684031597	NEW HARBINGER PUB	$16.95		pregnancy, parenting, fatherhood	adult
9781440856082	PRAEGER	$37.00		breast cancer, African American population	adult
9781422242759	MASON CREST	$25.95		LGBTQ+ health, teens	young adult
9780738220246	DA CAPO	16.99		Self-Help/ Borderline Personality Disorder	Adult
9780190610555	OXFORD UNIVERSITY PRESS	$24.95		back pain, aging	older adults
9781451621389	SIMON & SCHUSTER	16		Mental Health	Adult
9781442242623	ROWMAN & LITTLEFIELD	$39.00		breast cancer	adult
9780190677053	OXFORD UNIVERSITY PRESS	$21.95		disease prevention, breast cancer	adult
9781557049438	NEWMARKET PRESS	$9.95		heart disease, Latinx population, Hispanic population	adult
9781557049414	NEWMARKET PRESS	$9.95		diabetes, Latinx population, hispanic population	adult
9781421420103	JOHNS HOPKINS UNIV PRESS	$18.95		anxiety, parenting guide for children	adult
9781785925665	JESSICA KINGSLEY	$18.95		autism, women, graphic novel	adult

ISBN	Publisher	Price USD	Link	Topic/Audience	Age range
9781849053594	Jessica Kingsley Publishers	$9.95	http://www.worldcat.org/oclc/810119692	mental health	juvenile
9781843102069	Jessica Kingsley Publishers	$12.56	http://www.worldcat.org/oclc/53937841	mental health, disability	juvenile
9781849054645	Jessica Kingsley Publishers	$9.95	http://www.worldcat.org/oclc/858901492	disability	juvenile
9781849052979	Jessica Kingsley Publishers	$14.95	http://www.worldcat.org/oclc/823085228	aging, dementia, mental health	juvenile
9781849055635	Jessica Kingsley Publishers	$14.95	http://www.worldcat.org/oclc/909387245	mental health, depression	juvenile
9781849054690	Jessica Kingsley Publishers	$13.95	http://www.worldcat.org/oclc/861207358	chronic illness, diabetes	juvenile
9781849059527	Jessica Kingsley Publishers	$8.54	http://www.worldcat.org/oclc/830992850	learning disabilities	juvenile
9781849054478	Jessica Kingsley Publishers	$9.95	http://www.worldcat.org/oclc/864789888	disability	juvenile
9781849053099	Jessica Kingsley Publishers	$9.95	http://www.worldcat.org/oclc/877431454	disability, epilepsy	juvenile
9781849059480	Jessica Kingsley Publishers	$13.95	http://www.worldcat.org/oclc/827777731	degenerative disease, aging, disability	juvenile
9780226306643	UNIV OF CHICAGO PRESS	$25.00		cancer	adult
9780944235959	American Cancer Society	$15.70	http://www.worldcat.org/oclc/172979798	cancer	adults/ children

ISBN	Publisher	Price USD	Link	Topic/Audience	Age range
9780007254682	HarperCollins Publishers	$17.36	http://www.worldcat.org/oclc/123113327	cancer	adults
9781118592052	JOHN WILEY	$22.99		cancer nutrition	adult
9780198719854	OXFORD UNIVERSITY PRESS	$21.95		cancer prevention	adult
9780375714740	pantheon	$16.95		cancer, graphic novel	adult
9788466628082	EDICIONES B	$29.95		cancer, graphic novel	adult
9781608198061	BLOOMSBURY	$28.00		caregiving, aging parents, personal narrative, graphic novel	adult
9781421433578	JOHNS HOPKINS UNIV PRESS	$54.95		caregiving, aging parents	adult
9780300207989	YALE UNIVERSITY PRESS	$20.00		caregiving, alzheimers, dementia	adult
9780190259358	OXFORD UNIVERSITY PRESS	$19.95		caregiving, autism, parent guide to caring for children	adult
9780190259358	OXFORD UNIVERSITY PRESS	19.95		Autism	Adult
9781984817709	Bantam Books trade paperback edition	$21.60	http://www.worldcat.org/oclc/1128870823	pediatrics	adults
9781580406130	AMERICAN DIABETES ASSN	$18.95		nutrition, cuban cookbook, caribbean cookbook, diabetes	adult
9781782702115	AWARD PUBNS LTD	$6.43		nutrition, children	young adult
9781421423753	JOHNS HOPKINS UNIV PRESS	$22.95		pharmacy, children's medicines	adult

ISBN	Publisher	Price USD	Link	Topic/Audience	Age range
9781442251618	ROWMAN & LITTLEFIELD	$50.00		chronic illness, teens	young adult
978-0767921688	Broadway Books	28.49		Cookbook, Healthy meals	Adult
9780199734160	Oxford University Press	$16.80	http://www.worldcat.org/oclc/740965911	clinical trials, patient engagement	adults
				Good health habits - taking medicine, potty training, washing, sleeping.	Pre-K
9781138201118	ROUTLEDGE	$39.95		anxiety, depression, pregnancy	adult
				This bilingual series teaches care of the body. Translated by doctors, giving it professional authority, the series focuses on healthy practices.	K–Grade 2
	Capstone Press.			Addresses an element of a healthy lifestyle, including—physical hygiene, dental hygiene, exercise, and eating well. the books model decision-making in everyday life situations	K–Grade 2
9788478089406	Málaga Sirio D. L	$15.00	http://www.worldcat.org/oclc/870149373	complementary /alternative medicine	adults

ISBN	Publisher	Price USD	Link	Topic/Audience	Age range
9781462538546	GUILFORD	$16.95		addiction, family caregivers	adult
9781580406840	AMERICAN DIABETES ASSN	$18.95		diabetes, nutrition, cookbook	adult
9781580406628	AMERICAN DIABETES ASSN	$29.95		diabetes, nutrition, cookbook	adult
9780312332709	ST MARTIN'S PRESS	$21.95		pregnancy, fiction, African American population, teens	young adult
9781421412535	JOHNS HOPKINS UNIV PRESS	$24.95		chronic pain	adult
9781849059862	JESSICA KINGSLEY	$24.95		autism, book for parents to read with children	children
9781508187462	ROSEN YOUNG ADULT	$38.47		teens, bipolar disorder	young adult
9781725341258	ROSEN YOUNG ADULT	$18.24		LGBTQ+ health, teens	young adult
9781508187301	ROSEN YOUNG ADULT	$18.24		STDs, HIV, AIDs, teens	young adult
9780786449743	McFarland	39.95	https://www.amazon.[illegible]	PTSD	Adult
9781725341296	ROSEN YOUNG ADULT	$38.47		STDs, teens	young adult
9781557537607	PURDUE UNIVERSITY PRESS	$25.00		Alzheimers, caregiving	adult
9780525559252	PENGUIN BOOKS	$28.00		pregnancy, parenting, budgeting	adult

ISBN	Publisher	Price USD	Link	Topic/Audience	Age range
9780807014486	BEACON	$24.95		LGBTQ+ health, young women, Latinx population, Hispanic population, personal narrative	adult
9780813584867	RUTGERS UNIVERSITY PRESS	$42.95		fatherhood, children, fitness, health	adult
9781725309722	POWERKIDS PR	$23.60		teens, alcohol	young adult
9781682820223	REFERENCEPOINT PR INC	$30.95		teens, drug use	young adult
9781725309845	POWERKIDS PR	$23.60		teens, opioids, drug use	young adult
9781725309883	POWERKIDS PR	$23.60		teens, prescription drug use	young adult
9781725309920	POWERKIDS PR	$23.60		teens, tobacco use	young adult
9781725309968	POWERKIDS PR	$23.60		teens, tobacco use	young adult
9781551527659	ARSENAL PULP PRESS	$17.95		postpartumn depression, graphic medicine	adult
9781551525921	ARSENAL PULP PRESS	$26.95		cookbook, Latinx population, Hispanic population, nutrition	adult
9781442231917	ROWMAN & LITTLEFIELD	$40.00		dementia, alzheimers, caregiving	adult, older adults
9781442254282	ROWMAN & LITTLEFIELD	$42.00		veterans, family relationships	adults
9781442255821	ROWMAN & LITTLEFIELD	$39.00		depression, older adults	older adults
9781580406734	AMERICAN DIABETES ASSN	$19.95		diabetes, cookbook, nutrition	adults

ISBN	Publisher	Price USD	Link	Topic/Audience	Age range
9781119272243	JOHN WILEY	$22.99		breast cancer	adults
9781119363248	JOHN WILEY	$22.99		diabetes, keeping fit	adults
9781580405669	AMERICAN DIABETES ASSN	$16.95		diabetes	adults
9781580406123	AMERICAN DIABETES ASSN	$16.95		diabetes	adults
9781119967187	wiley-BLACKWELL	$37.50		diabetes, emotional aspects	adults
9781580405553	AMERICAN DIABETES ASSN	$16.95		diabetes, cookbook, nutrition	adult
9781580406215	AMERICAN DIABETES ASSN	$16.95		diabetes, Latinx population, Hispanic population	adult
9781421426488	Johns Hopkins Uni Press	$22.95		diabetes	adult
9781580405430	AMERICAN DIABETES ASSN	$16.95		diabetes, cookbook, nutrition	adult
9781580406796	AMERICAN DIABETES ASSN	$19.95		diabetes, cookbook, nutrition	adult
9781491465851	CAPSTONE PR INC	$6.95		diabetes, nutrition, children	children
9781580406635	AMERICAN DIABETES ASSN	$17.95		diabetes, caregiving	adult
9781508185918	ROSEN YOUNG ADULT	$12.95		disabilities, sexual health, teens	young adult
9780061582448	Collins	17.99		Guidebook/ Selfhelp/ Panic Attacks/ mental health	Adult
9781538727478	GRAND CENTRAL PUB	$20.99		prostate cancer	adult

ISBN	Publisher	Price USD	Link	Topic/Audience	Age range
9781620107911	LIMERENCE PR	$19.99		teens, sexual health, sexuality, pregnancy	young adult
9781849057370	jessica kingsley	$22.95		dyslexia, parents for children	adults
9781785922992	jessica kingsley	$18.95		dyslexia, children	children
9781534139343	CHERRY LAKE PUB	$12.79		children, nutrition	child
9780307888044	AMERICAN HEART ASSOCIATION	$16.00		heart disease, cookbook, nutrition	adult
9781465451521	DORLING KINDERSLEY, INC.	$12.99		children, nutrition	children
9781628321074	creative paperbacks inc	$9.99		children, nutrition, African American population	child
9781419712173	AMULET BOOKS	$12.99		disability, deafness, children, graphic novel	children, ages 8-11
	Heinemann Library			Exercise, how body parts are used to practce sport, and why sport is good for the body	K–Grade 2
9781101969809	Vintage Español, una división de Penguin Random House LLC	$12.88	http://www.worldcat.org/oclc/995325570	patient engagement	adults
9781440841019	PRAEGER	$37.00		lung cancer	adult
9780307594037	ALFRED A KNOPF	$25.00		cancer, end of life, personal narrative	adult
9781413325294	NOLO	$49.99		health system navigation	adults, older adults

ISBN	Publisher	Price USD	Link	Topic/Audience	Age range
9780740768576	Andrews McMeel Pub	$28.28	http://www.worldcat.org/oclc/134991818	cancer	adults
9780399592065	RANDOM HOUSE	$26.00		colon cancer, personal narrative	adult
9781508183426	ROSEN YOUNG ADULT	$12.95		teen, sexual health, birth control	young adult
9781508183518	ROSEN YOUNG ADULT	$12.95		tobacco, teens	young adult
9781508183617	ROSEN YOUNG ADULT	$34.45		teen, sexual health, birth control	young adult
9781462529025	GUILFORD	$16.95		pregnancy, depression prevention	adult
9781849055024	JESSICA KINGSLEY	$19.95		aspergers, depression	adult
9780271078465	PENN STATE UNIV PRESS	$24.95		miscarriage, pregnancy, sexual health, graphic novel, feminism	adult
9780812997392	Random House	27		Austism memoir	Adult
9780190622220	OXFORD UNIV PRESS US	$35.00		law, mental illness, caregiving	adult
9781482405668	GARETH STEVENS PUB LEARNI	$8.15		cookbook, nutrition, children	children
9780300190618	YALE UNIVERSITY PRESS	$20.00		cancer, caregiving, patients	adult
9780553536515	DELACORTE	$18.99		anxiety, teens, fiction	young adult
9781421418346	Johns Hopkins	$16.95		depression, women	adult

ISBN	Publisher	Price USD	Link	Topic/Audience	Age range
9780190888084	Oxford University Press	$7.29	http://www.worldcat.org/oclc/1045192902	palliative care, aging, end-of-life	adults
9781785922589	JESSICA KINGSLEY	$24.95		Transgender experience, graphic novel, LGBTQ+	adult
9780374156114	ST MARTIN'S PRESS	$26.00		menopause, personal narrative	older adults and adults
9780880919562	ACAD OF NUTR & DIETETICS	$17.99		nutrition, healthy aging, exercise	older adults
9781442235731	ROWMAN & LITTLEFIELD	$50.00		food allergies, nutrition, teens	young adult
9781554690671	ORCA	$9.95		Children, nutrition, graphic novel	children
9781452167916	Chronicle	$16.99		autism, fiction, children	children, ages 5-6
9780345536938	Bantam Books Trade Paperbacks	$10.69	http://www.worldcat.org/oclc/1104671039	complementary/alternative medicine	adults
9781939594259	HARRINGTON PARK PRESS	$45.00		prostate cancer, LGBTQ+	adult, older adults
9781626252974	NEW HARBINGER PUB	$17.95		gender identity, LGBTQ+, pregnancy, sexuality, teens	young adult
9780778736462	CRABTREE PUB CO	$8.95		nutrition, cookbook, children	children
9780778734079	CRABTREE PUB CO	$8.95		nutrition, cookbook, children	children
9781634256513	AMER BAR ASSOCIATION	$19.95		retirement, health, health navigation	older adults
9781462524938	GUILFORD	$14.95		attention deficit disoder, parent guide for children with ADHD	adult

ISBN	Publisher	Price USD	Link	Topic/Audience	Age range
9780545540629	GRAPHIX	$10.99		cystic fibrosis, siblings, children, graphic novel	children, ages 8-11
9780385346214	CLARKSON POTTER	$22.50		nutrition, cookbook, heart disease	adult
9781574415780	UNIV OF NORTH TEXAS PRESS	$21.95		nutrition, gluten free, cookbook	adult
9781849056700	Jessica Kingsley	$24.95		autism, mental health	adult
9781421417547	JOHNS HOPKINS UNIV PRESS	$19.95		ovarian cancer	adult
9781616141493	PROMETHEUS	$19.00		anxiety, shame	adult
9780545852500	GRAPHIX	$12.99		IBS, stress, children, graphic novel	children, ages 9-11
	Gareth Stevens Publishing			Each book, featuring several unnamed, racially diverse, and different abled children, introduces the body part, what it does and how to keep that body part clean to stay healthy	PreK–Grade 1
9780190846596	OXFORD UNIVERSITY PRESS	$29.95		mental health, LGBTQ+, PERSONAL NARRATIVE	ADULT
9781119283973	JOHN WILEY	$16.95		nutrition, pregnancy, infants	adult
9781541526808	LERNERCLASSROOM	$8.99		children, nutrition	children
9781541526815	LERNERCLASSROOM	$8.99		children, nutrition	children

ISBN	Publisher	Price USD	Link	Topic/Audience	Age range
9781632904850	CANTATA LEARNING	$7.95		children, nutrition	CHILDREN
9781450432788	HUMAN KINETICS	$17.95		heart disease	adults
9781499464443	ROSEN YOUNG ADULT	$12.95		drug use, teens	young adult
9781499464481	ROSEN YOUNG ADULT	$12.95		alcohol, teens	young adult
9781462527106	GUILFORD	$16.95		self-harm, guidebook for parents of teens	adult
9781626254657	NEW HARBINGER PUB	16.95		Anxiety in Adolescence	Adult
9781626254657	NEW HARBINGER PUB	$16.95		anxiety, parenting guide for teens	adult
9781684030989	new harbinger	$17.95		learning disabilities, parent guide for children	adults
9780545902489	SCHOLASTIC PRESS	$14.99		addiction, teens, graphic novel	young adult
9780062797186	Harper Wave	$10.99	http://www.worldcat.org/oclc/1099585117	patient engagement	adults
9780778723523	CRABTREE PUB CO	$7.95		nutrition, children	children
9781785927461	JESSICA KINGSLEY	$19.95		LGBTQ+, gender identity	adult, young adult
9781534563728	LUCENT BOOKS	$19.99		vaccinations, STDs, sexual health, teens, children	young adult
9781515739876	CAPSTONE PR INC	$6.95		nutrition, children, African American population	children

ISBN	Publisher	Price USD	Link	Topic/Audience	Age range
9780190494636	OXFORD UNIVERSITY PRESS	$12.95		attention deficit disorder, parent guide for children with ADHD	adult
9781482409697	GARETH STEVENS PUB LEARNI	$8.15		food allergies, nutrition, children	children
9781482409758	GARETH STEVENS PUB LEARNI	$8.15		food allergies, nutrition, children	children
9781632280671	CLEIS	$16.95		sexual health, teens	young adult
9781580405997	AMERICAN DIABETES ASSN	$19.95		diabetes, nutrition, Asian population, cookbook	adult
9781501709951	ILR CORNELL	$19.95		patient engagement, navigating hospitals	adult
9781580406604	AMERICAN DIABETES ASSN	$18.95		diabetes management	adult
9780763668723	CANDLEWICK PRESS	$12.99		puberty, sexual health, preteen, teens	young adult
9780763774288	JONES & BARTLETT LEARNING	$29.95		colon cancer	adult
9781426218651	National Geographic Society	22.99		Health and fitness	Adult
9781442221826	Rowman & Littlefield		http://www.worldcat.org/oclc/844461226	pediatrics	adults
9781438004839	BARRON'S	$9.99		child anxiety, parenting guide	adult

ISBN	Publisher	Price USD	Link	Topic/Audience	Age range
9781785927164	JESSICA KINGSLEY	$17.95		autism children, music, parenting guide	adult
9781580406819	AMERICAN DIABETES ASSN	$21.95		cookbook, diabetes, Hispanic population, Latinx population, nutrition	adult
9781557048547	NEWMARKET PRESS	$15.95		health, Latinx population, Hispanic population	adult
9781534408944	ATHENEUM CHILDREN'S BOOKS	$17.99		anxiety, children, starting school	children
9781941302415	roar	$19.99		eating disorders, teens, graphic medicine	young adult
	The Rosen Publishing Group.			describes how and why a child should engage in daily care of the body whether that is eating well, exercising, brushing one’s teeth or brushing one’s hair.	K–Grade 2
9781682827451	REFERENCEP OINT PR INC	$30.95		nutrition, teens, African American population	young adult
9780199465880	OXFORD UNIVERSITY PRESS	$19.95		heart disease, aging, nutrition, exercise	older adults
9781422235065	MASON CREST	$23.95		LGBTQ+ health, teens	young adult
9781421411514	The Johns Hopkins University Press	$12.85	http://www.worldcat.org/oclc/944099240	aging, safety	adults
9781534563698	LUCENT BOOKS	$19.99		attention deficit, teens	young adult

ISBN	Publisher	Price USD	Link	Topic/Audience	Age range
9781534564824	LUCENT BOOKS	$19.99		allergies, teens	young adult
9781421422336	JOHNS HOPKINS UNIV PRESS	$22.95		cancer	adult
9780740753855	ANDREWS MCMEEL PUBLISHING	$9.99		veterans, disability, graphic novel	adults
9780062319814	HARPERCOLLINS	$25.99		aging, heart disease, chinese-american population	older adult
9781413325539	NOLO	$29.99		health system navigation	older adults
	Combel Editorial			good nutrition and oral hygiene.	PreK–K
9781421422282	JOHNS HOPKINS UNIV PRESS	$18.95		dementia, alzheimers, caregiving	older adult, adult
9781442266155	rowman and littlefield	$39.00		cancer, caregiving, patients	adult
9781284150278	JONES & BARTLETT LEARNING	$20.95		lung cancer	adult
9781421426679	JOHNS HOPKINS UNIV PRESS	$19.95		dementia, caregiving, end of life	older adult, adult
9780415625159	ROUTLEDGE	$30.95		menopause	adult, older adults
9780199336920	OXFORD UNIVERSITY PRESS	$23.95		prostate cancer	older adults
9780199330041	OXFORD UNIVERSITY PRESS	$20.95		chronic pain, parent guide for children	adults
9781421409467	johns hopkins	$33.00		depression	adults

ISBN	Publisher	Price USD	Link	Topic/Audience	Age range
9781421410562	Johns Hopkins University Press	$22.57	http://www.worldcat.org/oclc/864532950	aging, men's health	adults
9781592407323	GOTHAM BOOKS	$20.00		depression, graphic novel	adults
9781978803480	RUTGERS UNIVERSITY PRESS	$34.95		sexual health, marriage, health, LGBTQ+	adults
9781641702348	FAMILIUS	$12.99		attention deficit, children, mindfulness	children
9781328662057	Scribe Publications	28		Therapy/mental health/ psychology	Adult
978-1893005488	Mayo Clinic Press	$16.20		children's health	adults
978-1893005600	Mayo Clinic Press	$9.99		pregnancy	adults
9781911171539	FLYING EYE BOOKS	$17.95		children, anxiety, fiction	children
9781119625445	JOHN WILEY	$22.99		health system navigation	older adults
9781119348870	JOHN WILEY	$22.99		medicare, health system navigation	older adults
9781938870064	HEALTH PROF PAUL BROOKES	$44.99		dementia, memory, communication	older adults
9780062447289	HarperOne	17.99		Womens Health/ Menopause	Adult
9781848192744	jessica kingsley	$19.95		menopause	adults, older adults
9780271087122	PENN STATE UNIV PRESS	$29.95		menopause, graphic novel	older adults, adults
9781580401760	amer diabetes assn	$19.95		diabetes, cookbook, nutrition, Latinx population	adults
	Picture Window Books.			Disabilites including neurodiversity	K–Grade 2

ISBN	Publisher	Price USD	Link	Topic/Audience	Age range
9781580407106	amer diabetes assn	$16.95		stress, diabetes, health	adults
9781626254893	NEW HARBINGER PUB	$16.95		Stress management	young adult, adults
	capstone press			Food portions and nutrition	K–Grade 2
9780810958401	HARRY N ABRAMS	$12.95		cancer, caregiving, graphic novel	adult
9781580406901	AMERICAN DIABETES ASSN	$16.99		cookbook, diabetes, nutrition	adults
9780292745490	UNIV OF TEXAS PRESS	$24.95		cookbook, diabetes, nutrition, Latinx population, Hispanic population	adults
9780763786830	Jones and Bartlett Publishers	$19.69	http://www.worldcat.org/oclc/754987548	cancer	adults
9780199945719	OXFORD UNIVERSITY PRESS	$20.95		STROKE	ADULT
9780553447187	HARMONY CROWN	$35.00		heart disease, cookbook, nutrition	adult
9781580406758	amer diabetes assn	$17.95		diabetes, cookbook, nutrition, African American population	adult
9781626727717	ROARING BROOK	$17.99		disabilites, children, education, picture book	children ages 5-8
	Picture Window Books.			How the senses function.	K–Grade 2

ISBN	Publisher	Price USD	Link	Topic/Audience	Age range
9781581103212	American Academy of Pediatrics	$10.83	http://www.worldcat.org/oclc/555620148	nutrition, pediatrics	adults
9781421422497	JOHNS HOPKINS UNIV PRESS	$22.95		obesity prevention	adults
9781481489188	SIMON & SCHUSTER BOOKS	$18.99		ocd, mental health, teens	young adults, ages 12 and up
9780385537322	DOUBLEDAY	$25.00		teen pregnancy, fiction, African American population	young adult
9780762754427	GLOBE PEQUOT	$18.95		veterans, mental health, health navigation	adults
9781422236079	MASON CREST	$23.95		teens, drug use	young adults
9781580895910	CHARLESBRIDGE PUBLISHING	$7.99		nutrition, children	children
9781848192645	JESSICA KINGSLEY	$12.95		chronic pain, graphic novel	adult
9781433815713	AMER PSYCHOLOGICAL ASSN	$18.95		adhd, parenting guide	adult
9781538102091	Rowman & Littlefield	$35.00	http://www.worldcat.org/oclc/1004121660	patient engagement	adults
9780763774295	JONES & BARTLETT LEARNING	$24.95		cancer, older adults	older adults
9781477758076	POWERKIDS PR	$8.25		food allergies, children	children
9781580406024	amer diabetes assn	$19.95		diabetes, cookbook, nutrition	adults
9781422236093	MASON CREST	$23.95		drug use, teens	young adult
9781581109818	AMER ACAD OF PEDIATRICS	$19.95		nutrition, children, parent guide	adult
9781472947789	Bloomsbury	20		Exercise Therapy	Adult

ISBN	Publisher	Price USD	Link	Topic/Audience	Age range
9780553384628	bantam	17		Parenting bipolar child	Adult
9781580406741	AMERICAN DIABETES ASSN	$16.95		prediabetes, cookbook, nutrition	adult
9781442243026	rowman and littlefield	$50.00		teen pregnancy, parenting	young adult
9780780815575	omnigraphics	$69.00		teen pregnancy, parenting	young adult
9781118944233	JOHN WILEY	$22.99		heart disease	adults
9780190674984	New York, NY : Oxford University Press	$24.95	https://www.worldcat.org/title/preventing-medication-errors-at-home/oclc/1083591780&referer=brief_results	Drugs, prescriptions, medication errors	adults
9781538110768	ROWMAN & LITTLEFIELD	$36.00		sexual health, social health, lgbtq+, teens	young adults
9781849056557	JESSICA KINGSLEY	$17.95		fiction, child, depression	children
9781608192786	BLOOMSBURY	$18.00		mental illness, graphic novel	adults
9781433811340	AMERICAN PSYCHOLOGICAL AS	$12.95		children, attention deficit	children
9781848192539	jessica Kingsley	$24.95		dementia, aging, chinese american population	older adults
9780970124517	Institute for Healthcare Advancement	$12.95	http://www.worldcat.org/oclc/838556311	pediatrics	adults
9780970124555	Institute for Healthcare Advancement	$12.95	http://www.worldcat.org/oclc/854564263	aging	adults

ISBN	Publisher	Price USD	Link	Topic/Audience	Age range
9780970124531	Institute for Healthcare Advancement	$12.95	http://www.worldcat.org/oclc/270947214	teen health	adults
9780972014854	Institute for Healthcare Advancement	$12.95	http://www.worldcat.org/oclc/841379045	pediatrics, obesity	adults
9780972014816	Institute for Healthcare Advancement	$12.95	http://www.worldcat.org/oclc/724512926	dental	adults
9780307407610	CLARKSON POTTER	$26.00		cookbook, nutrition, heart disease	adult
9781620105863	LIMERENCE PR	$9.99		gender identity, lgbtq+, teens, graphic novel	young adult
9781620106945	LIMERENCE PR	$9.99		disabilities, sexual health, graphic novel	adults
9780385670388	Anchor Canada	21		Aspergers memoir	Adult
9780803232532	UNIV OF NEBRASKA PRESS	$26.95		cookbook, nutrition, fitness, indigenous american population	adult
9781609805166	SEVEN STORIES PRESS	$23.95		cancer, graphic novel	adult
9781936303564	demos	$19.95		menopause	adults and older adults
9781455598540	GRAND CENTRAL PUB	$28.00		mental health, medication, graphic novel, personal narrative	adults
9781760295097	ALLEN & UNWIN	$22.95		backpain	adults
9781421419244	johns hopkins	$18.95		STDs, sexual health	adults

ISBN	Publisher	Price USD	Link	Topic/Audience	Age range
9781442230569	rowman and littlefield	$34.00		sexual health, veterans	adults
9781442275089	rowman and littlefield	$35.00		sexual health, cancer	adults
9780780813854	OMNIGRAPHICS	$69.00		sexual health, teens, African American population	young adults
9780199595655	OXFORD UNIVERSITY PRESS	$22.95		STDs, sexual health	adults
9781501193255	ATRIA BOOKS	$26.00		anxiety, African American population, males	adults
9781937163259	IN FACT BOOKS	$15.95		mental illness, personal narrative	adults
9781640637320	ENTANGLED TEEN	$17.99		teen, fiction, health	young adults, 15-18
9780740791963	ANDREWS MCMEEL	$9.99		veterans, tbi, graphic novel	adults
9781534564886	LUCENT BOOKS	$19.99		teen, cancer	young adults
9781534564794	LUCENT BOOKS	$19.99		teen, skin conditions	young adults
9780545132060	GRAPHIX	$10.99		dentistry, teens, graphic novel	young adults
9781432991241	Heinemann Library	$8.95		children, healthy snacking, nutrition	children
9781413325874	NOLO	$29.99		medicare, health system navigation	older adults
9781606997604	FANTAGRAPHICS BOOKS	$22.99		aging, graphic medicine	older adults
9781785924347	JESSICA KINGSLEY	$22.95		AUTISM , WOMEN	ADULTS

ISBN	Publisher	Price USD	Link	Topic/Audience	Age range
9781626728585	ROARING BROOK	$16.99		SPEECH ANXIETY, FICTION, CHILDREN, LATINX, HISPANIC	CHILDREN, AGES 8-9
9781422236116	MASON CREST	$23.95		teens, drug use	young adults
9781462519859	GUILFORD	$17.95		medications, children, mental health	adults
9781421426297	JOHNS HOPKINS UNIV PRESS	$19.95		depression	adults
9781462507894	Guilford	19.95		ADHD/ Parental Guide	Adult
9781433817472	aMER PSYCHOLOGICAL ASSN	$16.95		anxiety, worry, stress	adults
9781442248656	Rowman & Littlefield Publishers	$5.98	http://www.worldcat.org/oclc/951141434	doctor-patient communication	adults
9781616086398	W W NORTON	$14.95		alzheimers, caregiving, graphic medicine	adults
9781433820991	aMER PSYCHOLOGICAL ASSN	$16.95		attention deficit, parent guide for teens and children, African American population	adults
9781682825075	rEFERENCEPOINT PR INC	$30.95		distracted driving, teens	young adults
9781682827550	rEFERENCEPOINT PR INC	$30.95		tobacco, teens	young adults
9781580406659	AMERICAN DIABETES ASSN	$19.95		diabetes, nutrition, cookbook	adults
9780262029124	MIT PRESS	35		Death/ Loss	Adult
9780547636450	Houghton Mifflin Harcourt	30.95		Autism Memoir	Adult

ISBN	Publisher	Price USD	Link	Topic/Audience	Age range
9781607745648	Ten Speed Press, Random House Inc	$16.99	http://www.worldcat.org/oclc/877909109	cancer	adults
9780393710632	W.W. Norton & Company	$17.20	http://www.worldcat.org/oclc/880565999	mental health	adults
9780911910988	Merck Sharp & Dohme Corp	$17.95	http://www.worldcat.org/oclc/860903323	health reference	adults
9780911910308	Merck Research Laboratories	$39.95	http://www.worldcat.org/oclc/1104897007	health reference	adults
	Vintage	$17.32	http://www.worldcat.org/oclc/993944572	mental health, depression	adults
9781558328822	Harvard Common Press	$17.99	http://www.worldcat.org/oclc/1041228441	pediatrics, breastfeeding	adults
9780385352284	Alfred A. Knopf	$22.46	http://www.worldcat.org/oclc/907967998	patient engagement	adults
9780399184772	Nielsen	25		Autoimmune cookbook	Adult
9780271071121	PENN STATE UNIV PRESS	$32.95		lung cancer, graphic medicine	adults
9781250088550	FLATIRON BOOKS	$25.99		transgender children, fiction, LGBTQ+ health	young adult, adults
9781935864448	oncology nursing society	$23.00		cancer, teens	young adult
9781580406208	AMERICAN DIABETES ASSN	$18.95		diabetes	adults

ISBN	Publisher	Price USD	Link	Topic/Audience	Age range
9780786469468	Mcfarland	$35.00		PTSD, Veterans	adults
9781442223233	ROWMAN & LITTLEFIELD	$51.00		lung cancer, caregivers, patients	adults
9781440857201	praeger	$37.00		weight loss, nutrition	adults
9781538122235	ROWMAN & LITTLEFIELD	$30.00		caregiving	adults
9781616147518	PROMETHEUS	$20.00		dementia, caregiving	adults
9781476776231	SIMON & SCHUSTER	$26.00		Autism, parenting guide for children	adults
9781626251205	NEW HARBINGER	$18.85		DEPRESSION	ADULTS
9781512425307	TWENTY-FIRST CENTURY	$37.32		vaccination, teens	young adults
9780231153072	Columbia University Press	$12.06	http://www.worldcat.org/oclc/635465362	pediatrics, vaccines	adults
9781482405767	GARETH STEVENS PUB LEARN	$8.15		nutrition, children, cookbook	children
9780472052196	UNIV OF MICHIGAN PRESS	$24.94		genetics, women, cancer, personal narratives, gentic testing	adults
9780393081916	w w norton	$26.95		genetics, Populations - hispanic, latinx, indigenous americans	adults
9780740762024	ANDREWS MCMEEL PUBLISHING	$9.99		veterans, disability, graphic novel	adults

ISBN	Publisher	Price USD	Link	Topic/Audience	Age range
9781508117254	ROSEN CLASSROOM	$6.33		children, nutrition, grocery shopping	children
9781935826040	KALINDI PR	$9.95		children, nutrition	children
9781580405584	amer diabetes assn	$17.95		diabetes, nutrition	adults
9781534128682	CHERRY BLOSSOM PR	$11.36		children, nutrition	children
9780972014809	Institute for Healthcare Advancement	$12.95	http://www.worldcat.org/oclc/958934430	dental	adults
9780970124548	Institute for Healthcare Advancement	$12.95	http://www.worldcat.org/oclc/912138982	aging	adults
9780972014892	Institute for Healthcare Advancement	$12.95	http://www.worldcat.org/oclc/868039620	teen health	adults
9780970124500	Institute for Healthcare Advancement	$12.92	http://www.worldcat.org/oclc/918234292	pediatrics	adults
9780972014861	Institute for Healthcare Advancement	$11.66	http://www.worldcat.org/oclc/1045798942	pediatrics, asthma	adults
9780972014847	Institute for Healthcare Advancement	$12.95	http://www.worldcat.org/oclc/922341366	pediatrics, obesity	adults
9780970124562	Institute for Healthcare Advancement	$12.95	http://www.worldcat.org/oclc/1045798508	pregnancy	adults
9781523501502	Workman	27.5		Pregnancy	Adult
9780761181507	Workman	16.95		Parenting/ Infants/ Toddlers	Adult
9780761152774	Workman	15.95		Parenting/ Infants/ Toddlers	Adult

ISBN	Publisher	Price USD	Link	Topic/Audience	Age range
9780761152149	Workman	16.95		Parenting/ Infants/ Toddlers	Adult
9780399580239	TEN SPEED	$16.99		nutrition, infants	adults
9780763636326	CANDLEWICK PRESS	$16.99		nutrition, children	children
9781433823084	aMER PSYCHOLOGICAL ASSN	$19.95		attention deficit, adults, caregiving	adults
9780062656476	HARPERCOLLINS	$17.99		autism, fiction, teens, relationships	young adult
9781771642156	Greystone Books	17.95		Dementia/Caregiving	Adult
9781421420141	JOHNS HOPKINS UNIV PRESS	$35.00		depression, caregiving	adults
9781421420646	JOHNS HOPKINS UNIV PRESS	$39.95		dementia, caregiving, end of life	adults and older adults
9780684834078	SIMON & SCHUSTER	16.99		Depression/Caregiving	Adult
9781849056489	JESSICA KINGSLEY	$22.95		Puberty, sexual health, parenting guide, children, teens, autism, intellectual disabilities	adults
9780300204650	YALE UNIVERSITY PRESS	$22.00		chronic pain, children, parenting guide	adults
9781785927287	JESSICA KINGSLEY	$18.95		children, LGBTQ+ health	children
9781847808653	FRANCES LINCOLN	$12.99		children, nutrition	children
9781541526839	LERNERCLASSROOM	$8.99		children, nutrition	children
9781541526846	LERNERCLASSROOM	$8.99		children, nutrition	children
9781541526853	LERNERCLASSROOM	$8.99		children, nutrition	children

ISBN	Publisher	Price USD	Link	Topic/Audience	Age range
9781541526860	LERNERCLASSROOM	$8.99		children, nutrition	children
9781632171795	LITTLE BIGFOOT	$18.99		teens, sexual health, sexuality, pregnancy, puberty	preteens and young adults
9781612542287	BROWN BOOKS PUB GROUP	$14.95		children, nutrition	children
9780300184020	YALE UNIVERSITY PRESS	$18.00		healthy aging	older adults
9781421424200	johns hopkins	$19.95		heart disease, women	adults
9781442251960	ROWMAN & LITTLEFIELD	$34.00		veterans, health system, disability	adults
9781250151025	FEIWEL & FRIENDS	$19.99		eating disorders, teens, body image, indigenous american population	ages 9-11 and young adults
9780531213100	CHILDRENS PRESS	$9.95		children, nutrition, hydration	children
9781421423517	johns hopkins	$24.95		IBS, Caregiving, parents to children	adults
9781580405645	AMERICAN DIABETES ASSN	$17.95		diabetes	adults
