# Supplementary material for: Facilitating rural access to quality health information through Little Free Libraries
Source: J Med Libr Assoc. 2023 Oct 2;111(4):811–8. doi: 10.5195/jmla.2023.1585 (PMC10621719; doi:10.5195/jmla.2023.1585)
Supplement: Supplementary file 2 — Appendix B: Online Resources for Printing and Ordering [file jmla-111-4-811-s02.pdf]

Appendix B: Online Resources for Printing and Ordering

Title	Authors/ Organization	Year updated	Format
YOUR HEALTHIEST SELF Disease Prevention Checklist	NIH		2 page handout
Questions to ask before you take your medicine	AHRQ		3 page handout
YOUR HEALTHIEST SELF Social Wellness Checklist	NIH		3 page handout
All of Us Research Program Bookmark	NLM	2019	Card
Because an informed community is a healthier community, Bookmark	ALA		Card
Because Libraries are Partners in a Healthy Community, Bookmark	ALA		Card
Medication Wallet Card	AHRQ		Card
MedlinePlus Bookmark English and Spanish	NLM	2019	Card
What is the All of Us Research Program?	NLM	2019	thicker paper
Your Family Your History Your Health Discover Your Story, Rackcard	NLM	2018	thicker paper
Do you know the right questions to ask?	AHRQ		double sided paper
How Can I Keep Track of Physical Activity and Healthy Eating?	American Heart Association		double sided paper

Title	Authors/ Organization	Year updated	Format
Trust it or trash it?	Genetic alliance		double sided
BECAUSE THE RIGHT HEALTH QUESTIONS CAN LEAD TO BETTER HEALTH CARE.	ALA		Poster size for display at LFL
BECAUSE HEALTH AND EDUCATION GO HAND IN HAND.	ALA		Poster size for display at LFL
BECAUSE LIBRARIES ARE PARTNERS IN A HEALTHY COMMUNITY.	ALA		Poster size for display at LFL
BECAUSE YOUR FAMILY HEALTH HISTORY MATTERS.	ALA		Poster size for display at LFL
https://thatsaclaim.org/health-primary-school/	Informed Choices Network		Poster size for display at LFL
Eat Right Food, Nutrition and Health Tips from the Academy of Nutrition and Dietetics	Academy of Nutrition and Dietetics	2020	sheet of paper, double sided
Make Smart Choices! Nutrition Label Word Search puzzle	FDA	2018	single sheet of paper
The Nutrition Facts Label Tip Card	FDA	2019	single sheet of paper
Tome decisiones inteligentes!/ Nutrition Label Word Search puzzle	FDA	2018	single sheet of paper
Energize Your Body with Fruits and Vegetables!	California Department of Public Health	2016	single sheet of paper, double sided
Smart Tips for Getting Nutrients That May Be Lacking	the Academy of Nutrition and Dietetics	2020	single sheet of paper, double sided
All of Us Research Program	NLM	2019	single sheet paper
Reliable Sources of Health Information/ Recursos de información de salud para usted	NLM	2018	single sheet paper

Title	Authors/ Organization	Year updated	Format
Your Community<br>Your World<br>Your Discovery<br>Explore Your Inner Scientist	NLM	2018	single sheet paper
Your Data<br>Your Device<br>Your Apps<br>Use Technology for Your Health	NLM	2018	single sheet paper
Your Family Your History Your Health/ Su Familia Su Historia Su Salud	NLM	2018	single sheet paper
Your Family<br>Your History<br>Your Health<br>Discover Your Story/ Su Familia<br>Su Historia<br>Su Salud<br>Descubra Su Historia	NLM	2018	single sheet paper, double sided
YOUR HEALTHIEST SELF<br>Physical Wellness Checklist	NIH		single sheet paper, double sided
Your Lifestyle<br>Your Wellness<br>Your Choice<br>Make the Best Decisions for You	NLM	2018	single sheet paper, double sided
Medications tracking	NIH		single sided
NLM 4 Caregivers	NLM		single sided
Be More Involved<br>in Your Health Care:<br>Tips for Patients	AHRQ		trifold
Lo que USTED puede hacer para prevenir caidas (What YOU Can Do to Prevent Falls - STEADI Spanish brochure)	CDC	2018	trifold

Title	Authors/ Organization	Year updated	Format
Participe más en su atención médica: Consejos para pacientes	AHRQ		trifold
MedlinePlus Basics	NLM	2019	trifold paper
MedlinePlus Basics Spanish	NLM	2019	trifold paper
Substance Misuse and Addiction Resources	NLM	2020	trifold paper

Link	Topic	Age range	Language
https://www.nih.gov/sites/default/files/health-info/wellness-toolkits/disease-[illegible]	disease prevention, health screenings, vaccinations	adults, older adults	English
https://www.ahrq.gov/sites/default/files/wysiwyg/patients-consumers/diagnosis-treatment/treatments/safemeds/your	Questions to ask doctor	adults, older adults	English
https://www.nih.gov/sites/default/files/health-info/wellness-toolkits/social-wellness-checklist.pdf	social wellness	adults, older adults	English
https://nnlm.gov/sites/default/files/sea/AllofUs/All%20of%20Us%20Bookmark.pdf	All of us	Adult	English
https://nnlm.gov/sites/default/files/sea/AllofUs/Informed%20Community%20Bookmarks.pdf	Public awareness, libraries and health	N/A	English
https://nnlm.gov/sites/default/files/sea/AllofUs/Libraries%20Are%20Partners%20Bookmarks.pdf	Public awareness, libraries and health	N/A	English
https://www.ahrq.gov/sites/default/files/wysiwyg/patients-consumers/diagnosis-treatment/treatments/safemeds/walletform.pdf	medications	adults, older adults	English
https://nnlm.gov/sites/default/files/sea/images/medlineplusbookmark.jpg	General consumer health	Adult	English and Spanish
https://nnlm.gov/sites/default/files/sea/AllofUs/All%20of%20Us%20Infographic%20Printready.pdf	All of Us, precision medicine	Adult	English
https://nnlm.gov/sites/default/files/sea/AllofUs/All_of_Us_RackCard_181219.pdf	Genetics	Adults	English
https://www.ahrq.gov/sites/default/files/wysiwyg/patients-consumers/patient-involvement/preguntas/10questionsEspanol.pdf	Questions to ask doctor	adults, older adults	English and Spanish
https://www.heart.org/-/media/data-import/downloadables/2/2/4/pe-abh-how-can-i-keep-track-of-physical-activity-and-eating-ucm_300471.pdf	fitness, nutrition	adults, older adults	English

Link	Topic	Age range	Language
http://www.trustortrash.org/TrustorTrash.pdf	health information evaluation	adults	english
https://nnlm.gov/sites/default/files/sea/AllofUs/Better%20Questions%20Poster%2011x17.pdf	Public awareness, libraries and health	N/A	English
https://nnlm.gov/sites/default/files/sea/AllofUs/Health%20and%20Education%20Poster%2011x17.pdf	Public awareness, libraries and health	N/A	English
https://nnlm.gov/sites/default/files/sea/AllofUs/Libraries%20Are%20Partners%20Poster%2011x17.pdf	Public awareness, libraries and health	N/A	English
https://nnlm.gov/sites/default/files/sea/AllofUs/Family%20Health%20History%20Poster%2011x17.pdf	Public awareness, libraries and health, family health history	N/A	English
https://thatsaclaim.org/health-primary-school/	Critical thinking, evaluation	N/A	English
https://nnlm.gov/ZxL	nutrition, portion sizes, healthy snacking	adults	english
https://nnlm.gov/Zxo	nutrition, games	adults and children	english
https://nnlm.gov/ZxU	How to read a nutrition label	adults	english
https://nnlm.gov/Zx4	nutrition, games	adults and children	Spanish
https://nnlm.gov/ZxZ	nutrition, food serving size	adults	English and Spanish
https://nnlm.gov/Zxu	nutrition	adults and children	english
https://nnlm.gov/sites/default/files/sea/AllofUs/All%20of%20Us%20One%20Pager%20FAQ.pdf	All of Us	Adult	English
https://nnlm.gov/sites/default/files/sea/AllofUs/All_of_Us_Spanish_508_181219.pdf	nutrition, healthy eating, general consumer health information, healthcare navigation	children and adult	Spanish

Link	Topic	Age range	Language
https://nnlm.gov/sites/default/files/sea/AllofUs/All_of_Us_Citizen_Science_Flyer_181219.pdf	Environmental health, mobile health resources, citizen science	children and adult	English
https://nnlm.gov/sites/default/files/sea/AllofUs/All_of_Us_Technology_Resources_508_Flyer_181219.pdf	HIV/AIDs, childhood developmental milestones tracker, quitting smoking, traveling, health literacy trivia, herbal supplements, nutrition, fitness	children and adult	English
https://nnlm.gov/sites/default/files/sea/AllofUs/All_of_Us_Spanish_Flyer_181219.pdf	general consumer health, environmental health, healthcare system navigation, genetics, nutrition	children and adult	Spanish
https://nnlm.gov/sites/default/files/sea/AllofUs/All_of_Us_Eng_Span_Flyer_181219.pdf	Aging, Pillbox, healthcare system navigation, household products, complementary medicine, environmental health, nutrition, quitting smoking, healthy living, fitness, health information in different languages, genetics, MedlinePlus	children, adults, older adults	English and Spanish
https://www.nih.gov/sites/default/files/health-info/wellness-toolkits/physical-wellness-checklist.pdf	healthy weight, exercise	adults	English
https://nnlm.gov/sites/default/files/sea/AllofUs/All_of_Us_Health_and_Wellness_Flyer_181219.pdf	MedlinePlus, health information in different languages, wellness, fitness for older adults, healthy eating, HIV/AIDS, evaluating online health information	children, adults, older adults	English and Spanish
https://www.nia.nih.gov/sites/default/files/2018-05/medication-worksheet-contrast.pdf	medications	adults, older adults	English
https://sis.nlm.nih.gov/outreach/caregiversflyer.pdf	caregiver health	adults, parents	english
https://www.ahrq.gov/sites/default/files/publications/files/beinvolved.pdf	talking to your dr	adults	English
https://www.cdc.gov/steadi/spanish/pdf/patient/WhatYOUCanDo-Final-Spanish-508.pdf	fall prevention	older adults	Spanish

Link	Topic	Age range	Language
https://www.ahrq.gov/sites/default/files/publications2/files/beinvolvedsp.pdf	talking to your dr	adults	Spanish
https://nnlm.gov/sites/default/files/shared/files/trifolds/mptri.pdf	General consumer health	Adult	English
https://nnlm.gov/sites/default/files/shared/files/trifolds/mptri_esp.pdf	General consumer health	Adult	Spanish
https://nnlm.gov/sites/default/files/sea/files/Trifolds/NLM%20-%20Substance%20Misuse%20and%20Addiction%20Resources%2002-28-2020.pdf	Substance abuse, opioids	Adult	English
