# Supplementary figures and images for: Facilitating rural access to quality health information through Little Free Libraries
Source: J Med Libr Assoc. 2023 Oct 2;111(4):811–8. doi: 10.5195/jmla.2023.1585 (PMC10621719; doi:10.5195/jmla.2023.1585)

Appendix C: Average Response by Question

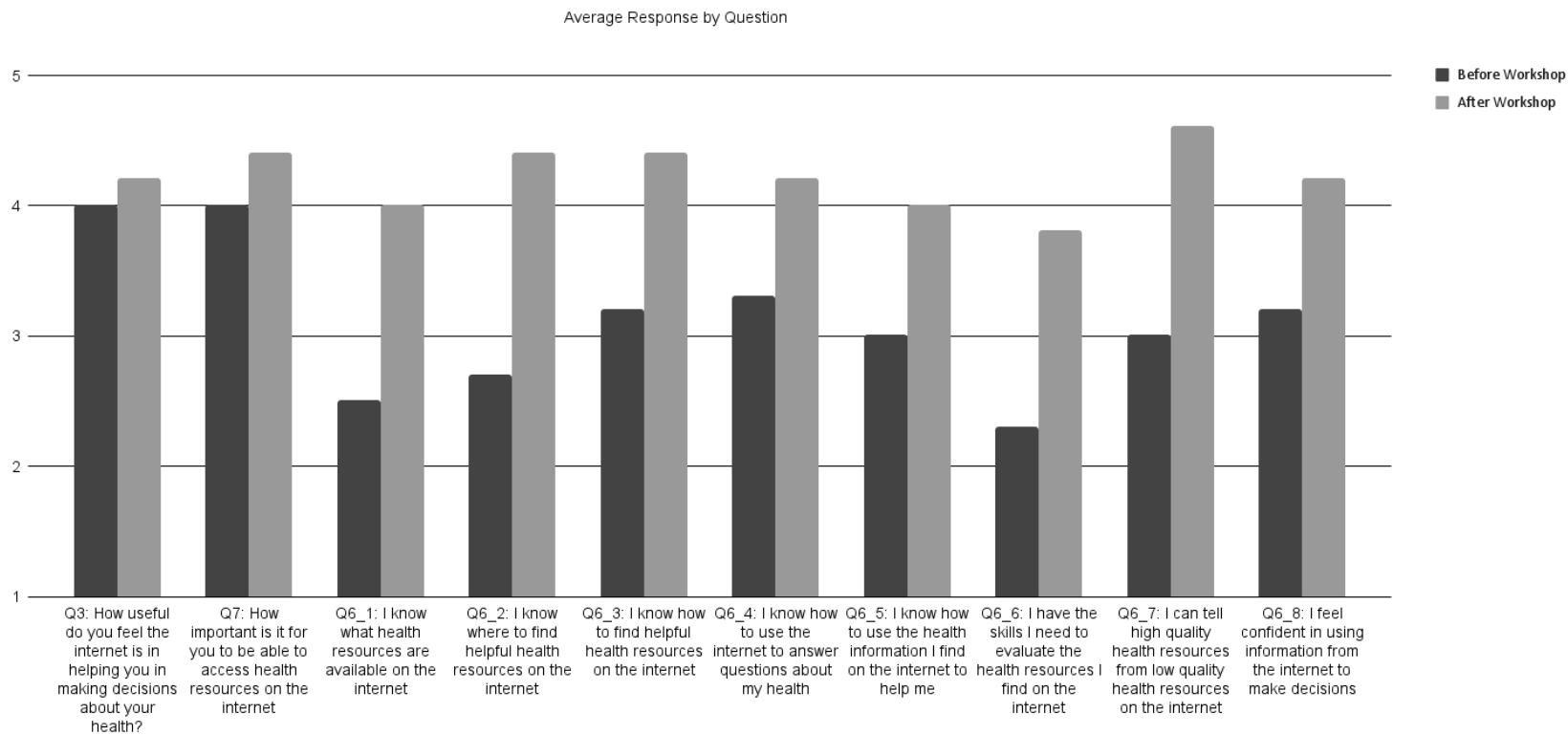

Supplement: Supplementary file 3 — Appendix C: Average Response by Question [file jmla-111-4-811-s03.pdf]
