# Supplementary material for: Facilitating rural access to quality health information through Little Free Libraries
Source: J Med Libr Assoc. 2023 Oct 2;111(4):811–8. doi: 10.5195/jmla.2023.1585 (PMC10621719; doi:10.5195/jmla.2023.1585)
Supplement: Supplementary file 4 — Appendix D: eHeals Results [file jmla-111-4-811-s04.pdf]

Appendix D: eHeals Results

DATE	TIME	Q1	Q3	Q7	Q6_1	Q6_2	Q6_3	Q6_4
After workshop								
6/17/2020	827am	yes	useful	important	agree	agree	agree	agree
6/15/2020	913am	yes	useful	very important	agree	strongly agree	strongly agree	agree
6/12/2020	328pm	yes	useful	very important	agree	strongly agree	agree	strongly agree
6/11/2020	219pm	yes	useful	important	agree	agree	agree	agree
6/10/2020	453pm	yes	very useful	important	agree	agree	strongly agree	agree
Before workshop								
6/17/2020	827am	yes	unsure	important	disagree	undecided	undecided	disagree
6/11/2020	217pm	yes	useful	important	agree	agree	agree	agree
6/10/2020	453pm	yes	useful	unsure	agree	undecided	agree	undecided
6/9/2020	210pm	yes	useful	important	disagree	disagree	agree	agree
6/9/2020	130pm	yes	useful	very important	disagree	agree	undecided	agree
6/9/2020	102pm	yes	useful	important	agree	agree	agree	agree
6/9/2020	1031am	yes	useful	important	undecided	disagree	undecided	undecided
6/9/2020	845am	yes	useful	important	disagree	disagree	disagree	disagree
6/8/2020	1032am	yes	useful	unsure	disagree	disagree	undecided	undecided

Q6_5	Q6_6	Q6_7	Q6_8
undecided	agree	strongly agree	undecided
agree	agree	strongly agree	strongly agree
strongly agree	agree	strongly agree	strongly agree
agree	agree	agree	agree
agree	undecided	agree	agree
undecided	disagree	undecided	disagree
agree	agree	undecided	undecided
undecided	disagree	disagree	undecided
agree	disagree	disagree	agree
disagree	disagree	afree	undecided
agree	undecided	undecided	undecided
disagree	disagree	undecided	agree
undecided	undecided	agree	undecided
undecided	disagree	disagree	disagree
